# Assessing Real-Time Moderation for Developing Adaptive Mobile Health Interventions for Medical Interns: Micro-Randomized Trial

**DOI:** 10.2196/15033

**Published:** 2020-03-31

**Authors:** Timothy NeCamp, Srijan Sen, Elena Frank, Maureen A Walton, Edward L Ionides, Yu Fang, Ambuj Tewari, Zhenke Wu

**Affiliations:** 1 Department of Statistics University of Michigan Ann Arbor, MI United States; 2 Molecular and Behavioral Neuroscience Institute University of Michigan Ann Arbor, MI United States; 3 Department of Psychiatry University of Michigan Ann Arbor, MI United States; 4 Addiction Center Department of Psychiatry University of Michigan Ann Arbor, MI United States; 5 Injury Prevention Center University of Michigan Ann Arbor, MI United States; 6 Department of Biostatistics University of Michigan Ann Arbor, MI United States; 7 Michigan Institute for Data Science University of Michigan Ann Arbor, MI United States

**Keywords:** mobile health, digital health, smartphone, mobile phone, wearable devices, ecological momentary assessment, depression, mood, physical activity, sleep, moderator variables

## Abstract

**Background:**

Individuals in stressful work environments often experience mental health issues, such as depression. Reducing depression rates is difficult because of persistently stressful work environments and inadequate time or resources to access traditional mental health care services. Mobile health (mHealth) interventions provide an opportunity to deliver real-time interventions in the real world. In addition, the delivery times of interventions can be based on real-time data collected with a mobile device. To date, data and analyses informing the timing of delivery of mHealth interventions are generally lacking.

**Objective:**

This study aimed to investigate when to provide mHealth interventions to individuals in stressful work environments to improve their behavior and mental health. The mHealth interventions targeted 3 categories of behavior: mood, activity, and sleep. The interventions aimed to improve 3 different outcomes: weekly mood (assessed through a daily survey), weekly step count, and weekly sleep time. We explored when these interventions were most effective, based on previous mood, step, and sleep scores.

**Methods:**

We conducted a 6-month micro-randomized trial on 1565 medical interns. Medical internship, during the first year of physician residency training, is highly stressful, resulting in depression rates several folds higher than those of the general population. Every week, interns were randomly assigned to receive push notifications related to a particular category (mood, activity, sleep, or no notifications). Every day, we collected interns’ daily mood valence, sleep, and step data. We assessed the causal effect moderation by the previous week’s mood, steps, and sleep. Specifically, we examined changes in the effect of notifications containing mood, activity, and sleep messages based on the previous week’s mood, step, and sleep scores. Moderation was assessed with a weighted and centered least-squares estimator.

**Results:**

We found that the previous week’s mood negatively moderated the effect of notifications on the current week’s mood with an estimated moderation of −0.052 (*P*=.001). That is, notifications had a better impact on mood when the studied interns had a low mood in the previous week. Similarly, we found that the previous week’s step count negatively moderated the effect of activity notifications on the current week’s step count, with an estimated moderation of −0.039 (*P*=.01) and that the previous week’s sleep negatively moderated the effect of sleep notifications on the current week’s sleep with an estimated moderation of −0.075 (*P*<.001). For all three of these moderators, we estimated that the treatment effect was positive (beneficial) when the moderator was low, and negative (harmful) when the moderator was high.

**Conclusions:**

These findings suggest that an individual’s current state meaningfully influences their receptivity to mHealth interventions for mental health. Timing interventions to match an individual’s state may be critical to maximizing the efficacy of interventions.

**Trial Registration:**

ClinicalTrials.gov NCT03972293; http://clinicaltrials.gov/ct2/show/NCT03972293

## Introduction

### Background

According to the World Health Organization, depression is the leading cause of disease-associated disability in the world [1]. In the United States, the burden of depression, including suicide, has continued to grow [2]. In populations at high risk, prevention of depression may be an effective strategy. The US National Academy of Medicine has highlighted the need to develop, evaluate, and implement prevention interventions for depression and other mental, emotional, and behavioral disorders [3].

Prevention interventions for depression are critical for individuals in stressful work environments because these environments can lead to increased rates of depression [4]. However, individuals in these work environments may have inadequate time or resources to access traditional mental health care services. High stress can also make individuals less receptive to interventions and behavior change [5,6].

Unlike other recent advances, mobile technology has the potential to transform the delivery and timing of depression prevention interventions to meet the needs of highly stressed individuals. In contrast to more intensive treatments (such as therapeutic appointments), mobile health (mHealth) interventions (such as push notifications) can be delivered at low burden, which may be critical given the individuals’ high stress workloads. Mobile devices hold the power to deliver just-in-time adaptive interventions (JITAIs) [7] to individuals during times when they are able to receive and respond to them. Mobile devices also collect objective measurements of an individual’s context and behavior with minimal burden (eg, step counts, sleep duration). These data may, in turn, be used to determine when to deliver interventions, and evaluate intervention efficacy, without bothering the individuals.

When initially designing a JITAI, these states of opportunity [7]—times when individuals are receptive to positive behavior change—are not known. Timing is critical because poorly timed interventions can lead to loss of engagement with the intervention [8]. Timing interventions is also particularly important for individuals in stressful work environments because poorly timed interventions could cause disengagement and treatment fatigue [9].

Current behavioral theories lack the granularity and adaptivity necessary to inform the timing of the delivery of mHealth interventions [10,11]. Many theoretical models are nondynamic—they only consider treatment adaptation based on baseline characteristics, such as sex and depression history [12]. Timing and adapting treatments based on real-time variables is essential for developing high-quality JITAIs [7].

This study follows a data-driven approach to inform the dynamic timing of intervention delivery. Experimentation and data collection were used to provide empirical evidence for determining states of opportunity—the data illustrate when interventions cause positive behavior change in individuals and when they do not.

There have been other empirical studies showing the promise of JITAIs to improve mental health [13]. Those studies are either focused on feasibility and acceptability of the JITAI [14-17] or use a randomized controlled trial (RCT) to demonstrate the impact of the JITAI on a distal outcome [18-20]. They do not focus on the timing of intervention delivery. In two studies [21,22], the authors demonstrated the benefits of timing mHealth intervention delivery based on real-time variables. In that work, the timing of intervention delivery is specified before the study. In contrast, because we did not know *a priori* how to time our interventions, our work used empirical evidence to learn how to dynamically time intervention delivery.

In statistical terms, we formulated the task of empirically learning how to dynamically time interventions as *discovering time-varying moderators of causal treatment effects* [23]. Time-varying moderators are *moderators* because they change—or moderate—the efficacy of subsequent treatments and are *time-varying* because the moderators’ values vary throughout the study (such as daily mood). For example, if push notifications containing sleep messages cause a larger increase in sleep when individuals had little sleep in the previous night compared with when individuals had high sleep, then the previous night’s sleep moderates the effect of sleep notifications. Discovering time-varying moderators informs treatment timing because treatment delivery can now be based on the observed values of these moderators. In the example, it may be better to send sleep notifications only after individuals have insufficient sleep. Note that time-varying moderators should have meaningful variability to allow the possibility of sending different interventions at different times.

We assessed time-varying moderators of mHealth interventions targeting 3 categories: mood, activity, and sleep. Stressful work environments can lead to sleep deprivation and physical inactivity [24-26], two behaviors directly associated with depression [24,27,28]. To prevent depression among individuals experiencing high stress, it is critical to develop high-quality interventions that can help them maintain and improve their mood, either through targeting mood directly or by indirectly improving activity and sleep [24,28].

Our study population comprised medical interns. Medical internship, the first year of physician residency training, is highly stressful, causing the depression rates of interns to be several folds higher than those of the general population [29]. Focusing on physician training, a rare situation in which a dramatic increase in stress can be anticipated, provides an ideal experimental model to develop interventions for maintaining mental wellness during life and work stressors.

Our study, the 2018 Intern Health Study (IHS) [30], was a 6-month-long mHealth cohort study that tracked medical interns using phones and wearables. During the internship year, we conducted a micro-randomized trial (MRT) [23]. Standard single–time point RCTs only inform moderation by baseline variables [31] and do not permit the discovery of time-varying moderators. The MRT was advantageous because it allowed us to discover time-varying moderators of causal treatment effects [23].

During each week in the 6-month study, an intern was randomized to 1 of 4 possible treatments: a week of mood notifications, activity notifications, sleep notifications, or no notifications. The outcomes were average daily self-reported mood valence (measured through a one-question survey), average daily steps (as a proxy for activity), and average daily sleep duration, where averages were 7-day averages of data collected during the week of treatment. The strongest moderators were hypothesized to be the previous week’s average daily mood, steps, and sleep, as these were the strongest predictors of the outcomes (based on previous years’ IHS data [30]). We were only interested in a subset of combinations of outcomes, treatments, and moderators.

### Study Aims

Here, we have highlighted the primary and secondary aims of this paper. Below, the *effect* (for which we are assessing moderation) corresponds to how a week of a certain notification category causally changes an outcome *compared with weeks of no notifications*.

The moderator aims listed below were not the only aims of the 2018 IHS. Analyses of main effects analyses were conducted before the analysis of moderator effects (see Additional Analyses in Multimedia Appendix 1). This paper has focused on moderator analyses as those were the most interesting findings.

#### Primary Aim

Our primary aim focused on discovering how an intern’s previous mood moderates the effect of notifications in general. Specifically, we examined the following: Is the effect of a week of notifications (of *any* category) on the average daily mood moderated by the previous week’s mood? Here, *Outcome*=mood; *Treatment*=any (mood, activity, or sleep); and *Moderator*=mood.

#### Exploratory Subaim

If we do find that mood moderates the effect of notifications, generally, we will assess if this moderation is consistent across all intervention categories. Specifically, we will examine the following: Is the effect of *each* category of notification on the average daily mood moderated by the previous week’s mood? Here, *Outcome*=mood; *Treatment*=mood, activity, and sleep separately; and *Moderator*=mood.

#### Secondary Aim 1

Secondary aim 1 focused on discovering how an intern’s previous activity moderates the effect of notifications containing activity messages. Specifically, we examined the following: Is the effect of a week of activity notifications on the average daily step count moderated by the previous week’s step count? Here, *Outcome*=steps, *Treatment*=activity, and *Moderator*=steps.

#### Secondary Aim 2

Secondary aim 2 focused on discovering how an intern’s previous sleep moderates the effect of notifications containing sleep messages. Specifically, we examined the following: Is the effect of a week of sleep notifications on the average daily sleep moderated by the previous week’s sleep? Here, *Outcome*=sleep; *Treatment*=sleep; and *Moderator*=sleep.

## Methods

### The Study App

Study participants were provided a Fitbit Charge 2 [32] to collect sleep and activity data, and a mobile app was downloaded to the intern’s phone. The app can conduct ecological momentary assessments (EMAs) [33], aggregate and visualize data, and deliver push notifications. The app was designed for iOS using Apple ResearchKit [34].

**Figure 1:**
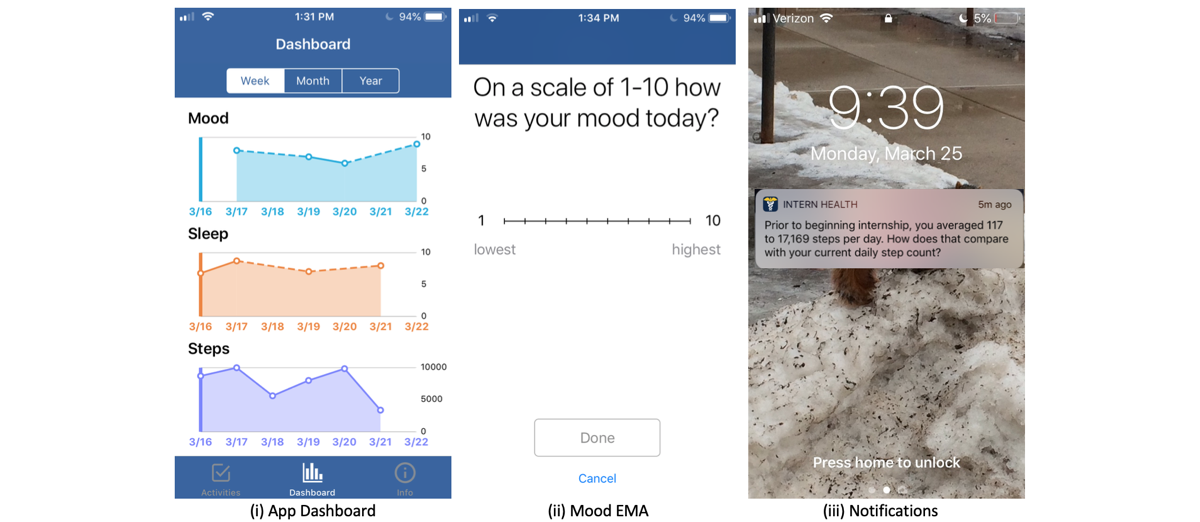
Screenshots of the app dashboard, mood ecological momentary assessment, and lock screen notifications.

As the primary aim of the study was focused on understanding the effects of interventions on the mental health of interns, we employed a daily EMA to measure mood valence (see Figure 1, Mood EMA). Daily mood is one of the 2 cardinal symptoms of depression [35]. This daily mood EMA is used widely to track the mood in patients with depression [36]. There are more widely used measurements of mental health other than mood valence (such as the Patient Health Questionnaire-9, PHQ-9 [37]). However, these questionnaires are more time-intensive and their assessment may cause higher nonresponse rates. Participants are prompted to enter their daily mood every day at a user-specified time between 5 PM and 10 PM.

In addition to collecting EMA data, the app aggregates and displays visual summaries of interns’ historical data, including step and sleep counts (collected through the Fitbit) and mood EMA data (Figure 1, App Dashboard). The data are integrated with the app using Fitbit’s publicly available application programming interface [38]. Displaying historical trends to the intern helps them self-monitor their mood, activity, and sleep trajectories and could potentially lead to positive reactive behavior change [39]. These displays are a type of *pull* intervention, that is, interventions that are available at all times but only accessed upon user request. The *pull* component was available to all participants, and assessing its effects was not the focus of this study.

The IHS app also delivers *push* interventions, that is, interventions delivered without user prompting. Evaluating and improving the delivery timing of the push notification intervention was the focus of this study.

### Push Notification Intervention

As applied to mHealth, theoretically, behavior change comprises an individual’s motivation and ability to change, combined with a trigger to elicit change [40]. Push notifications are such a trigger, potentially providing motivational messages for change (eg, to spark change), strategies for change (eg, to facilitate change), and/or reminders to engage with the app (eg, to signal change) [40]. Importantly, research supports the potential of push notifications for behavior change [41,42].

Push notifications are particularly advantageous for medical interns because they are delivered as needed with minimal burden to the user [42-44]. However, poorly timed push notifications can lead to loss of engagement and treatment fatigue [9,45], demonstrating the importance of evaluating and improving the delivery timing of the push notifications.

Push notifications were provided to the interns through the app, with the goal of improving healthy behavior in a target category of interest: mood, activity, and sleep (ie, mood notifications improve mood, activity notifications increase physical activity, and sleep notifications increase sleep duration). For all 3 categories, there were 2 types of notifications: tips and life insights. Consistent with theory [40] and motivational interviewing approaches [46-48], tips are non–data-based notifications that provide autonomy support (eg, motivational focused messages on why change) and tools (eg, ability-focused messages on how to change) to promote healthy mood, activity, or sleep. Next, consistent with theory [40,49,50] and research showing that interventions that enhance self-monitoring promote behavior change [51], life insight notifications summarize an individual’s data, to provide reminders (eg, signals) and/or reduce the burden of accessing the app to view visualizations. Table 1 contains examples of different push notifications used in the study.

**Table 1:** Examples of 6 different groups of notifications.

Notification groups	Life insight	Tip
Mood	Your mood has ranges from 7 to 9 over the past 2 weeks. The average intern’s daily mood goes down by 7.5% after intern year begins.	Treat yourself to your favorite meal. You’ve earned it!
Activity	Prior to beginning internship, you averaged 117 to 17,169 steps per day. How does that compare with your current daily step count?	Exercising releases endorphins which may improve mood. Staying fit and healthy can help increase your energy level.
Sleep	The average nightly sleep duration for an intern is 6 hours 42 minutes. Your average since starting internship is 7 hours 47 minutes.	Try to get 6 to 8 hours of sleep each night if possible. Notice how even small increases in sleep may help you to function at peak capacity & better manage the stresses of internship.

### The Intern Health Study Micro-Randomized Trial Design

**Figure 2:**
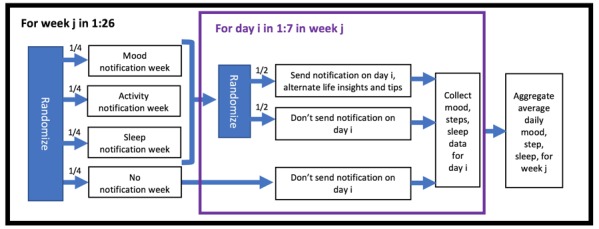
Randomization scheme of the Intern Health Study micro-randomized trial.

To discover time-varying moderators for informing the timing of notification delivery, we conducted an MRT. The MRT design is shown in Figure 2. The MRT design and protocol were approved by the University of Michigan Institutional Review Board (Protocol #HUM00033029).

The main randomization was the weekly randomization to a specific notification category (mood, activity, sleep) or to no notification. Thus, we were able to compare how a week of a certain notification category changed intern behavior when compared with a week of no notifications.

The randomization—and the ensuing analysis of effects—occurred at the weekly level for two reasons. First, the notifications are not intended to change the interns’ behavior in the next few hours, but over the next few days. Randomizing and analyzing effects at the weekly level, as opposed to a daily or minute level, permitted the discovery of longer-term effects. Second, as interns are quite busy, their behavior may not change significantly after receiving a single notification. Instead, interns received several notifications related to the same category and had a consistent reminder about improving that category.

Given a week when a user was randomized to receiving notifications, every day they were further randomized (with 50% probability) to receive a notification on that day. Hence, for a mood notification week, the user received, on average, 3.5 mood notifications that week. The purpose of this randomization was to balance delivering enough notifications to be noticeable and cause behavior change but not too often that it leads to treatment fatigue [9]. Treatment fatigue is pervasive in mHealth [7] and for individuals with heavy workloads [9]. Additional Analyses in Multimedia Appendix 1 contains a summary of how many notifications users received in a given week.

Another way to prevent treatment fatigue is through increased variability in notifications and the order in which they are received [52]. For each notification category, the notifications alternated between life insights and tips. In addition, given a type and category, each notification was drawn randomly, without replacement, from a corresponding bucket of notifications. The bucket refilled once it was completely emptied. Alternating between life insights and tips increased the day-to-day variability of the notification framing. Drawing notifications without replacement ensured that users were not receiving repeats of the same notification. Under this scheme, on average, a user did not receive a repeat notification for 16 weeks. Weekly and daily notification randomization and notification delivery were implemented using the Firebase Cloud Messaging platform [53].

### Participants

Medical doctors starting their year-long internship in the summer of 2018 were eligible to participate in the study. Interns were onboarded before the start of their internship (between April 2018 and June 2018), in which they were instructed to download the study app, were provided Fitbits, completed a baseline survey, and were able to begin entering mood scores. Baseline and follow-up surveys were administered through the app using Qualtrics survey software [54]. Data collection began when an intern enrolled in the study and continued until the end of the trial. Collecting data before the start of the internship provided baseline measurements of mood, step counts, and sleep, which are valuable control variables in the analysis. The weekly randomizations and notification delivery began on June 30, 2018, 1 day before the start of interns’ clinical duties. Interns were rerandomized every 7 days thereafter. During the study, notifications were sent at 3 PM, mood EMAs were collected daily between 5 PM and 10 PM, and sleep and step data were collected every minute. Data were transferred directly from the subjects’ phones to a secure, Health Insurance Portability and Accountability Act–compliant server managed by the University of Michigan Health Information and Technology Services. The interns received notifications for 6 months (26 weeks), and the trial ended on December 28, 2018.

### Statistical Analysis

#### Overview

To analyze the primary and secondary aims, we performed a moderator analysis for each of the outcomes, treatments, and moderators specified in Study Aims. More details on the methods can be found in Further Details on the Statistical Methods in Multimedia Appendix 1.

In the analysis, there were 4 sets of variables:

1. The *treatment outcome* variable of interest, Y_t_.
2. The *treatment indicator*, *Z*_t_. For now, *Z*_t_ is a binary indicator, where *Z*_t_=1 implies it is a notification week (of any category) and *Z*_t_=0 is a no-notification week. The case with multicategorical treatments—mood, activity, and sleep notifications—will be described under the secondary aims.
3. The *moderator*, *M*_t_, corresponding to the causal effect moderator of interest.
4. The last set of variables, *X*_t_, are the *control variables*. The control variables are variables measured before each weekly randomization (eg, baseline data and previous weeks’ data) and are included in the model to reduce variation in the outcome, *Y*_t_.

The outcomes, treatment, and moderators correspond exactly to the outcomes, treatments, and moderators described in Study Aims. As interns were randomized to different treatments each week, the outcomes, treatments, moderators, and control variables were aggregated at the weekly level and were indexed by time, *t*, corresponding to each week of the study (*t*=1,…,26).

To perform the moderator analysis, we used a linear model with an interaction term. The outcome of interest (such as average daily mood), *Y_t_*, was regressed on *X_t_*, *M_t_*, *Z_t_* and the interaction between *M_t_* and *Z_t_*, *Z_t_M_t_*, giving the model the following form:

E*(Y_t_|X_t_,M_t_,Z_t_)* = *a_0_X_t_ + a_1_M_t_ + b_0_Z_t_ + b_1_Z_t_M_t_*

The moderation effect of interest is the coefficient *b_1_* for the interaction of *Z_t_* and *M_t_*. This coefficient is interpreted as the change in treatment effect of treatment *Z_t_* on *Y_t_* per unit change in *M_t_*. A positive value for *b_1_* indicates that the treatment works better after weeks when *M_t_* is high, whereas a negative value indicates that the treatment works better after time points when *M_t_* is low.

For the primary and secondary aims, to evaluate if the moderator effect is statistically significant, we performed a hypothesis test comparing the coefficient *b_1_* to 0, with a 0.05 type I error rate. We reported the estimate of *b_1_*, the standard error, and *P* value of this test. Though estimating and testing the moderation effect is useful, it does not demonstrate whether the notifications had a positive or negative effect on the outcome. Hence, in addition to a hypothesis test, we also plotted the estimated treatment effect at various values of the moderator. We did this by using both the estimate of the slope, *b_1_*, and intercept, *b_0_*, of the moderation effect. The plots also included histograms of the moderator to illustrate the distribution of treatment effects.

#### Estimation Techniques

To estimate the coefficients, we used a multicategorical extension of the weighted and centered least-squares estimator described in Boruvka et al [55]. The estimation method provides asymptotically unbiased estimates of the causal effect moderation of interest. The method also protects against potential misspecification of terms not interacted with treatment (*a_0_X_t_ + a_1_M_t_*). The method assesses the uncertainty of the coefficient estimates using robust standard error estimation—the *sandwich* estimator [56]—to account for correlation between outcomes over time. The method was implemented in R using the package geepack [57]. The code is available on the first author’s website.

#### Missing Data

Missing data occurred throughout the trial because of interns not completing the self-reported mood survey or not wearing Fitbits. Multiple imputation [58], a robust method for dealing with missing data, was used to impute missing data at the daily level. Due to the complexity of the trial design and data structure, our imputation method combines imputation methods for longitudinal data [59] and sequentially randomized trials [60]. Results were aggregated across 20 imputed datasets using Rubin’s rules [58,61]. We also assessed the sensitivity of the conclusions to the imputation method. See Missing Data and Sensitivity Analyses in Multimedia Appendix 1 for further details on the missingness and sensitivity analysis results.

#### Primary Aim

The primary aim assessed the previous week’s average daily self-reported mood valence as a moderator of the effect of notifications on the average daily self-reported mood valence. For this analysis, the interpretation, *b_1_*, was *the change in treatment effect (for delivering a week of notifications compared with a week of no notifications) on the average daily mood when the previous week’s average daily mood increased by 1*.

#### Secondary Aim 1

The first secondary aim assessed the previous week’s average daily step count as a moderator of the effect of activity notifications on the average daily step count. For this aim, the treatment variable (*Z_t_*) and corresponding coefficients (*b_0_* and *b_1_*) were no longer binary because there were 4 possible notification categories. See Further Details on the Statistical Methods in Multimedia Appendix 1 for further details on the multicategorical treatment model. The focus of inference for secondary aim 1 was on the first dimension of the moderation effect, *b_11_*, which corresponds to the comparison between activity notification weeks and no-notification weeks. In addition, to reduce right skew and decrease outliers, the outcome and moderator used average daily *square root* step count. After the square root transformation, the average daily step counts more closely resembled a Gaussian distribution.

The interpretation, *b_11_*, was *the change in treatment effect (for delivering a week of activity notifications compared with a week of no notifications) on the average daily square root step count when the previous week’s average daily square root step count increased by 1*. Hypothesis testing was performed on *b_11_* and plots were made using estimates of *b_01_* and *b_11_*.

#### Secondary Aim 2

Secondary aim 2 assessed the previous week’s average daily sleep count as a moderator of the effect of sleep notifications on the average daily sleep count. Similar to secondary aim 1, the treatment here was no longer binary, and we encoded the treatment vector the same way as secondary aim 1. For this analysis, the focus of inference was on the second dimension, *b_12_*, which compared sleep notification weeks with no-notification weeks. Again, to reduce right skew and decrease outliers, the outcome and moderator used average daily *square root* sleep minutes.

The interpretation, *b_12_*, was *the change in treatment effect (for delivering a week of sleep notifications compared with a week of no notifications) on average daily square root sleep minutes when the previous week’s average daily square root sleep minutes increased by 1*. Hypothesis testing was performed on *b_12_*, and plots were made using estimates of *b_02_* and *b_12_*.

#### Exploratory Subaim

The exploratory aim assessed the previous week’s mood as a moderator of the effect of each notification category on the average daily mood. For the exploratory aim, the outcome and moderator were the same as the primary aim, except that the treatment was separated into 4 treatment categories (as in the secondary aims). As this aim was only exploratory, we did not calculate *P* values. Instead, for each notification category, we plotted the estimated treatment effect at various values of the moderator. This required making 3 separate lines using each dimension, with estimates of *b_0i_* providing the intercept and estimates of *b_1i_* providing the slope.

## Results

### Participants

Participants were recruited through emails, which were sent to future interns from 47 different recruitment institutions between April 1 and June 25, 2018. The recruitment institutions comprised both medical schools, where emails were sent to all graduates, and residency locations, where emails were sent to all incoming interns. A total of 5233 future interns received the initial email inviting them to participate in the study. In all, 40.78% (2134/5233) of interns downloaded the study app, completed the consent form, and filled out the baseline survey sometime before June 25, 2018. The study app and study participation were restricted to interns using an iPhone, the phone brand used by most interns. A total of 2134 interns received a Fitbit Charge 2. Of the 2134 interns, 1565 (73.34%) were randomly selected to participate in the MRT (see Additional Analyses in Multimedia Appendix 1 for an explanation of this initial randomization). These 1565 interns were randomized according to Figure 2. Interns were incentivized to participate in the study by receiving the Fitbit wearable and up to US $125, distributed 5 times throughout the year (US $25 each time) based on continued participation.

Of the 1565 interns in the MRT, 875 (55.91%) were female, and 774 (49.45%) had previously experienced an episode of depression. The interns represented 321 different residency locations and 42 specialties. The study interns’ baseline information closely resembled the known characteristics of the general medical intern population [29]. Throughout the trial, we measured intern mood valence, steps, and nightly sleep. Summaries of the weekly averages of those data can be found in Table 2.

**Figure 3:**
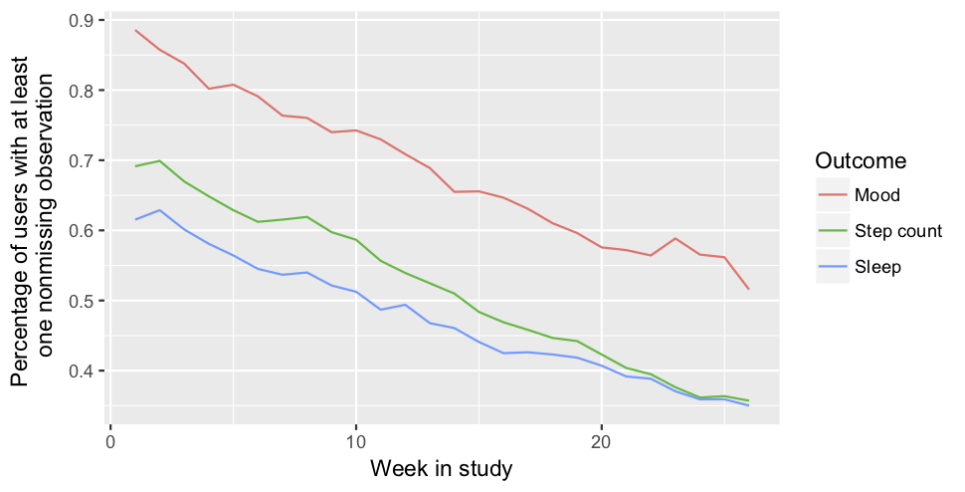
Percentage of interns with at least one nonmissing sleep, step, or mood observation for each week in the study.

Missing data occurred throughout the study. Figure 3 displays the percentage of interns with at least one nonmissing sleep, step, or mood observation for each week in the study. See Missing Data and Sensitivity Analyses in Multimedia Appendix 1 for further details on the missingness and sensitivity analyses.

**Table 2:** Summary statistics of daily mood, activity, and sleep during the study, averaged over each week of the study. These are the primary outcomes and moderators used in the analyses of all study aims.

Daily measure	First quartile	Median	Mean (SD)	Third quartile
Average daily mood	6.50	7.33	7.21 (1.43)	8.00
Average daily step count	6193	7983	8274 (3285)	10,050
Average daily hours of sleep	6.02	6.65	6.54 (1.25)	7.25

### Main Findings

#### Primary Aim

We conclude that the previous week’s average daily self-reported mood valence is a statistically significant negative moderator of the effect of notifications on the average daily self-reported mood valence. The estimate for the moderation is −0.052 (SE 0.014; 95% CI −0.081 to −0.023; *P*=.001).

**Figure 4:**
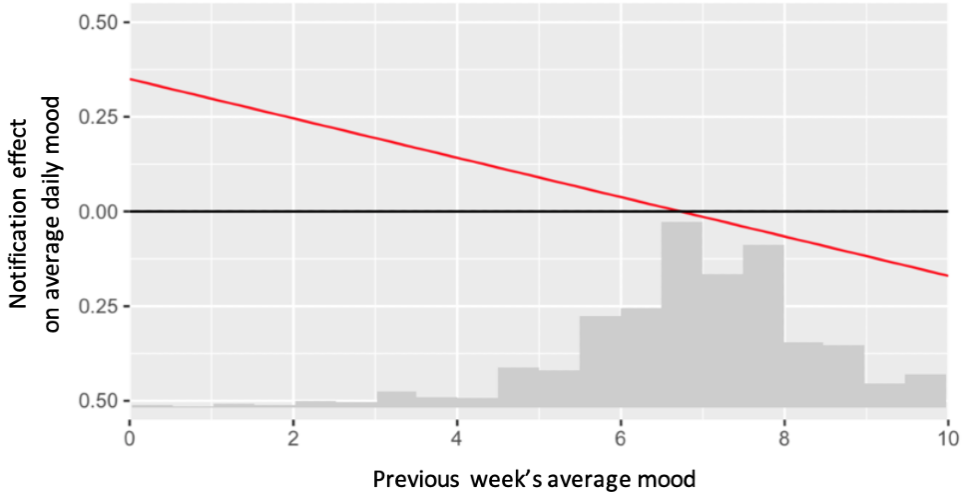
Estimated treatment effects (compared with no notifications) of notifications on average daily mood, at various values of previous week’s mood. The x-axis also contains a scaled histogram of previous week’s average mood.

Figure 4 plots the estimated treatment effect at various values of the moderator. Figure 4 shows that the effect of notifications (compared with no notifications) was positive for weeks when the previous mood was low, but negative for weeks when the previous mood was high. For example, when the previous week’s average daily mood was 3, we estimated that a week of notifications *increased* an intern’s average daily mood by 0.19 (effect size=0.14). However, when the previous week’s average daily mood was 9, we estimated that a week of notifications *decreased* an intern’s average daily mood by 0.12 (effect size=−0.08).

#### Exploratory Subaim

**Figure 5:**
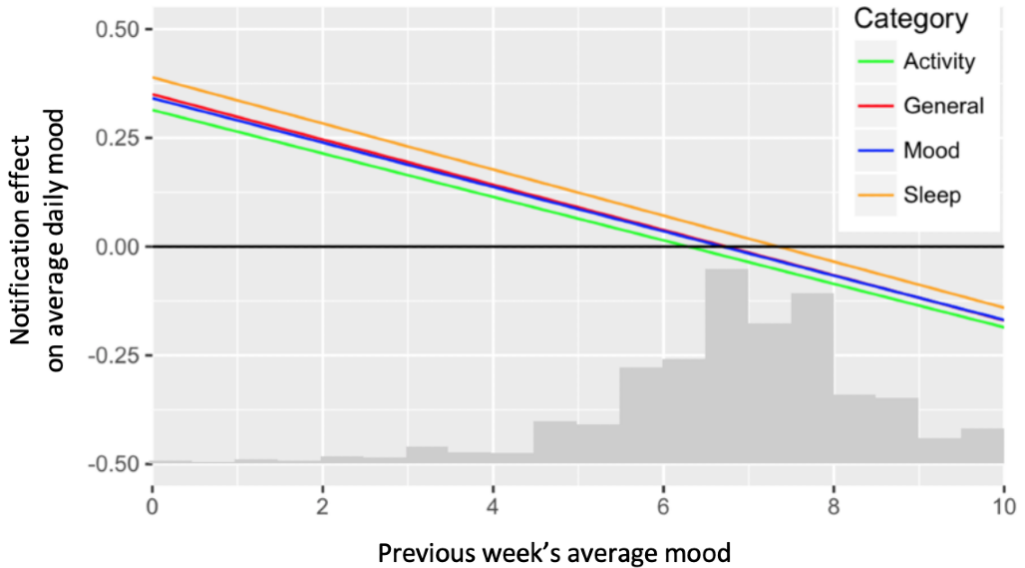
Estimated treatment effects (compared with no notifications) of different notification categories on average daily mood, at various values of previous week’s mood. The x-axis also contains a scaled histogram of previous week’s average mood.

For each notification category, we plotted the estimated treatment effect at various values of the moderator. Essentially, we broke apart the moderation effect in Figure 4 into 3 categories of notifications. The result is shown in Figure 5. We included the line for general notifications from Figure 4 for reference. Figure 5 demonstrates that the moderation by the previous week’s average daily mood was similar for all 3 notification categories.

When the previous week’s average daily mood was 3, we estimated that a week of mood, activity, and sleep notifications *increased* an intern’s average daily mood by 0.19, 0.16, and 0.23 (effect sizes=0.13, 0.11, and 0.16), respectively. When the previous week’s average daily mood was 9, we estimated that a week of mood, activity, and sleep notifications *decreased* an intern’s average daily mood by 0.12, 0.14, and 0.09 (effect sizes=−0.08, −0.10, and −0.06), respectively.

#### Secondary Aim 1

We conclude that the previous week’s average daily step count is a statistically significant negative moderator of the effect of activity notifications on average daily steps. The estimate for the moderation is −0.039 (SE 0.015; 95% CI −0.069 to −0.008; *P*=.01).

**Figure 6:**
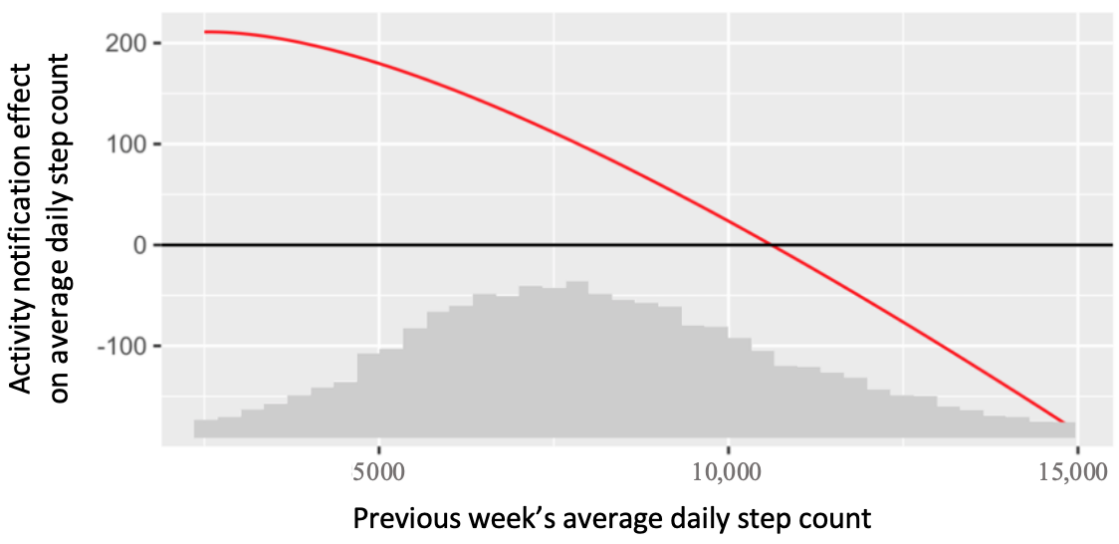
Estimated treatment effects (compared with no notifications) of activity notifications on average daily steps, at various values of previous week’s step counts. The x-axis also contains a scaled histogram of previous week’s average daily step count.

Figure 6 plots the estimated treatment effect at various values of the moderator. In Figure 6, for interpretability, we retransformed the moderation effect back from the analysis scale (square root) to the original scale. We see from Figure 6 that the effect of activity notifications (compared with no notifications) was positive for weeks when previous steps were low, but negative for weeks when previous steps were high. For example, when the previous week’s average daily step count was 5625, we estimated that a week of activity notifications *increased* an intern’s average daily step count by 165 steps (effect size=0.05). However, when the previous week’s average daily step count was 12,100, we estimated that a week of activity notifications *decreased* an intern’s average daily step count by 60 steps (effect size=−0.02).

#### Secondary Aim 2

We conclude that the previous week’s average daily sleep is a statistically significant negative moderator of the effect of sleep notifications on average daily sleep. The estimate for the moderation is −0.075 (SE 0.018; 95% CI −0.111 to −0.038; *P*<.001).

**Figure 7:**
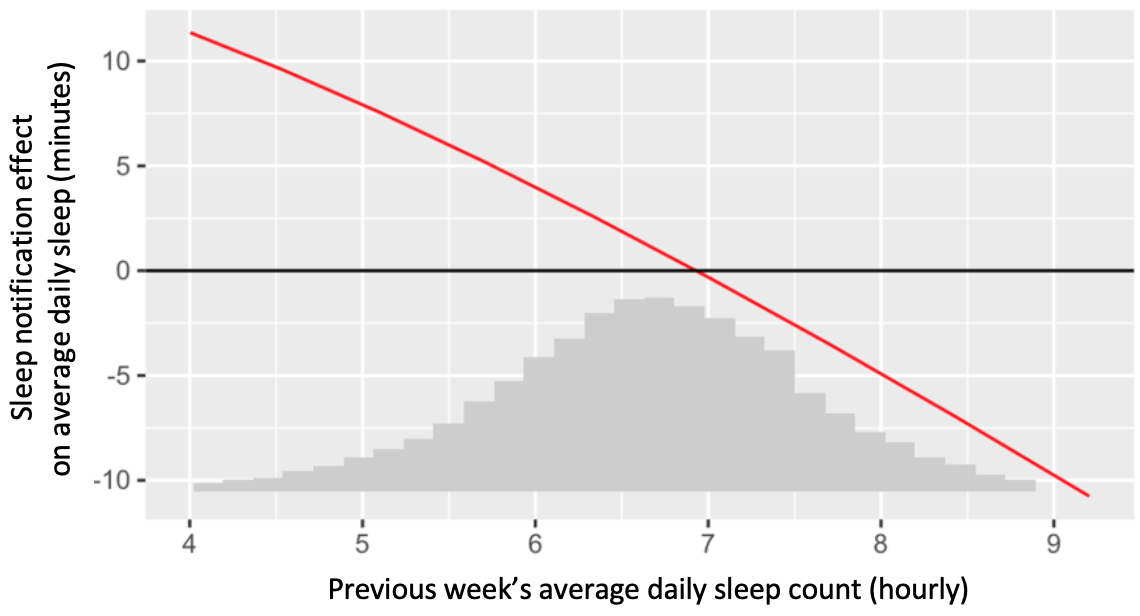
Estimated treatment effects (compared with no notifications) of sleep notifications on average daily sleep minutes, at various values of previous week’s hourly sleep. The x-axis also contains a scaled histogram of previous week’s average daily sleep count.

Figure 7 plots the estimated treatment effect at various values of the moderator. Again, we retransformed the moderation effect back from the analysis scale (square root) to the original scale. In addition, for interpretability, the x-axis is on the hourly scale, whereas the y-axis is on the minute scale. We see from Figure 7 that the effect of sleep notifications (compared with no notifications) was positive for weeks when previous sleep was low, but negative for weeks when previous sleep was high. For example, when the previous week’s average daily sleep was 5 hours, we estimated that a week of sleep notifications *increased* an intern’s average daily sleep by 8 min (effect size=0.11). However, when the previous week’s average daily sleep was 8 hours, we estimated that a week of sleep notifications *decreased* an intern’s average daily sleep by 5 min (effect size=−0.07).

### Additional Analyses

The Additional Analyses section of Multimedia Appendix 1 contains detailed results on other analyses, including an analysis of nonmoderated main effects, changes in effects over time, the effects of life insights and tips, the effects on long-term PHQ-9 scores, and an analysis of baseline moderators. There is evidence of a negative effect of (general) notifications on mood. There is also evidence of a positive effect of activity notifications on step count and a positive effect of sleep notifications on sleep duration. All of these effect sizes, however, are small. There is no strong evidence that these effects change over time. There is minor evidence that tips perform better than life insights in improving step count and sleep duration. We did not see any effects on long-term mental health outcomes. We saw some evidence of nonlinear moderation for the primary and secondary aims. The nonlinear moderator analysis suggested that when the moderators are high, the treatment effect on sleep hours and step count is close to 0 (as opposed to negative). Finally, we found that baseline variables, such as gender and depression history, were weak moderators of notification effects, demonstrating the value of personalizing intervention delivery on real-time data.

## Discussion

### Principal Findings

Through this MRT of an mHealth push notification intervention, we found that the effects of notifications were negatively moderated by the subject’s previous measurement of the outcome of interest. Specifically, we found that previous mood negatively moderated the effect of notifications on mood, previous step count negatively moderated the effect of activity notifications on step count, and previous sleep duration negatively moderated the effect of sleep notifications on sleep duration.

### Comparison With Other Studies

A few previous studies explored using real-time variables to determine the timing of mHealth interventions for mental health. These studies postulated that messages would be most effective when self-reported mood was outside the typical range [21], or when self-reported stress or negative affect was high [22]. The studies found that such timing does improve efficacy. Our work differs from these studies because we did not assume, beforehand, that interventions would be most effective during a predetermined time. Instead, we used the MRT design to *learn* opportune times to send interventions, based on real-time objective and self-reported data.

Outside of mental health, there have been studies that have sought to learn opportune times to send interventions. Much of that work is focused on assessing in-the-moment interruptibility, namely times when a user is open to interruption and willing to engage with a notification. For example, in one study [62], the authors found that phone usage, time of day, and location were strong predictors of a user’s willingness to engage with content provided via a push notification. Another study [63] found that location, affect, current activity, time of day, day of week, and current stress are significant predictors of a user’s willingness to respond to an EMA prompt. Another study [64] used an MRT to causally demonstrate that notifications (which ask users to self-monitor) are more effective when sent mid-day and on weekends. Our study differs from this work. In our study, the outcome was not focused on short-term engagement with the notification but rather longer-term behavior change, such as improved *weekly* mood, activity, or sleep.

Most standardized effect sizes within this paper fell within the 0.05 to 0.15 range. According to the suggested definitions of *small* and *large* [65], the effect sizes for our interventions are small. However, these definitions of *small* and *large* may not directly apply to the causal effects assessed in MRTs [66]. As MRTs are a relatively new trial design, there are currently no accepted definitions of *large* and *small* [66]. For the 3 MRTs with published effect sizes, the effects sizes were 0.074 [67], 0.2 [66], and 0.1 [66]. The effect sizes within this paper are similar in magnitude to these other works.

### Implications

Our principal findings demonstrate that the study interns’ current state meaningfully influences their receptivity to mHealth interventions for mental health. Effective mHealth interventions for individuals in stressful work environments must consider timing notification delivery based on recent real-time data. Delivering notifications when previous measurements of mood, sleep, and activity are low—when improvement is needed—benefits mood and behavior. However, delivering notifications when those variables are high, negatively impacts mood and behavior.

mHealth interventions aiming to increase mood, activity, and sleep can be improved based on these findings. An improved mHealth intervention for increasing mood would deliver notifications (of any type) only when the user’s previous week’s average daily mood is below 7 and sends nothing when previous mood is at or above 7. Similarly, for activity, an improved intervention would deliver activity notifications only when the user’s previous week’s average activity is below 10,614 steps and delivers nothing otherwise. For sleep, an improved intervention would deliver sleep notifications only when the user’s previous week’s average daily sleep duration is below 6.9 hours. These improved interventions are based upon our single trial, with small effect sizes. There is potential for larger effects through further intervention optimization and using different intervention groups in conjunction with each other. Consistent with the multiphase optimization strategy (MOST) framework [68,69], these suggested interventions should be further refined and evaluated in additional studies and confirmatory trials before being used broadly.

### Study Strengths

Through the MRT design and repeatedly randomizing interns throughout the trial, we were able to assess causal effect moderation by time-varying measurements. Our large sample size (1565 interns) allowed us to detect the moderators of interest. The relatively long duration of the study (6 months) demonstrated that our conclusions are valid beyond the first few weeks and months of the study (we analyzed how treatment effects vary over time in the Additional Analyses of Multimedia Appendix 1). Our study focused on medical interns, which provided a unique opportunity to assess the efficacy of mHealth interventions on wellness during life and work stressors. There were also advantages of our analytic approach. First, the use of the multicategorical extension of the weighted and centered least-squares estimator allowed us to unbiasedly assess the causal effect moderation. Second, our imputation method allowed us to cope with missing data without requiring strong assumptions.

### Limitations

The primary outcome for the study, mood valence, was self-reported. Self-reported outcomes may be less reliable and valid compared with objective measurements [70,71]. In addition, because of user nonresponse, missing data is a common issue with self-reported outcomes collected over an extended period [59]. In future studies, developing and using a passively collected objective measurement of depression could be beneficial for improving objectivity and reducing missing data.

The main findings of the IHS MRT are partially sensitive to the imputation method used for overcoming missing data (see Missing Data and Sensitivity Analyses in Multimedia Appendix 1). The conclusions of the primary aim and secondary aim 2 are not sensitive. The conclusion of secondary aim 1 (the negative moderation of the activity notification effect by previous step count), however, is sensitive to the imputation method.

The results of the IHS MRT may not extrapolate to other populations because medical interns are different from the general population in the average education level and socioeconomic status. Within the population of medical interns, sampling bias may still exist as the study’s interns self-selected into the study, as opposed to being randomly sampled. This self-selection bias may cause the study interns to be different from the general population of interns. For example, because they were motivated to participate in the study, they may also be motivated to change their behavior. Although it is difficult to show self-selection unbiasedness, the bias may be mitigated because a large percentage of interns agreed to participate in the study (40.78%), and the study interns’ baseline information closely resembles that of the general medical intern population [29].

Daily work schedules were not reliably measured in this study. Previous studies [63,64] have found that mHealth message effectiveness varies between weekdays and weekends, suggesting that future studies should assess work schedule as a potential moderator.

Measurements of app engagement could provide further insights into how these notifications are promoting behavior change. For example, after receiving a notification, users may have an increased rate of opening the app and viewing their historical data displays. Unfortunately, the app does not currently collect data on app access and app clicks. It also does not measure a user’s interactions with the notification messages. Including these capabilities in future versions of the app would be useful.

We did not have message tailoring in this study. Currently, the message framing and wording was the same, no matter the intern’s current behavior. The messages (see Table 1) are framed toward improving mood, sleep, and activity. This framing may be frustrating to an intern who already has a high mood or sufficient sleep or activity. Tailoring the wording of the messages [72,73] could potentially eliminate the negative effect of messages when previous mood, sleep, or activity is high (eg, providing a reminder message as opposed to an unnecessary ability-focused message).

There were also a couple technological errors that occurred throughout the trial. There were 8 days (of the 182 total days) when, because of server issues, no notifications were sent to any subject. In addition, the weekly randomization to a notification category occurred without replacement, as opposed to with replacement as originally intended.

### Future Iterations of the Intern Health Study

The IHS is an annual study that continues each year with a new cohort of interns [30]. Consistent with the MOST framework [68,69], this provides multiple trial phases to continually update, optimize, and test interventions and provides confirmation of findings from previous cohorts. Starting in the fall of 2019, we will run another study to test new hypotheses with improved interventions. Using the results and conclusions drawn from this study, in 2019, we plan to introduce tailored messages that are tailored based on an intern’s previous mood, activity, and sleep [72,73]. For people with high previous measurements, the messages will be framed toward maintenance of healthy behavior, not improvement. The cutoffs that define *high* and *low* scores will be based on data collected from the 2018 study. We also plan to improve the missing data protocol and incentive structure to reduce the frequency of missing data. We will collect work schedule information to compare message efficacy between work days and days off. Finally, in addition to providing notifications on the phone lock screen, we will also show the notifications within the app to give interns more opportunities to read them.

### Conclusions

Overall, our study demonstrates the importance of real-time moderators for the development of high-quality mHealth interventions, especially for individuals in stressful work environments. There were times when the notifications were beneficial and times when the notifications were harmful to the study participants. Developers of mHealth interventions are encouraged to think deeply about the delivery of interventions and how real-time variables can be used to inform the timing of intervention delivery. The MRT design allowed us to discover real-time moderators and may be useful for other app developers also aiming to learn when to deliver notification messages.

In addition to the research aims for future iterations of the IHS, assessing the value of mHealth interventions and delivery timing in other highly stressed populations is beneficial for understanding the generality of these results. Future MRTs should also examine the efficacy of mHealth content (eg, content focused on motivation, ability, or triggers) incorporated into other app features for behavior change. In this regard, developing mHealth intervention features beyond push notifications (eg, integrating ability-focused mindfulness exercises) could provide a greater overall benefit.

## Appendix

Multimedia Appendix 1 Additional analyses and statistical details.

Multimedia Appendix 2 CONSORT-eHEALTH Checklist (V 1.6.1).

## Abbreviations

EMA: ecological momentary assessment
IHS: Intern Health Study
JITAI: just-in-time adaptive intervention
MRT: micro-randomized trial
mHealth: mobile health
MOST: multiphase optimization strategy
NIH: National Institutes of Health
PHQ-9: Patient Health Questionnaire-9
RCT: randomized controlled trial
