# Supplementary material for: Assessing Real-Time Moderation for Developing Adaptive Mobile Health Interventions for Medical Interns: Micro-Randomized Trial
Source: J Med Internet Res. 2020 Mar 31;22(3):e15033. doi: 10.2196/15033 (PMC7157494; doi:10.2196/15033)
Supplement: Multimedia Appendix 1 [file jmir_v22i3e15033_app1.pdf]

Additional Analyses and Statistical Details for: Assessing Real-time Time Moderation for Developing Adaptive Mobile Health Interventions for Medical Interns: A Micro-randomized Randomized Trial

Timothy NeCamp Zhenke Wu, Elena Frank, Maureen A. Walton, Edward Ionides, Yu Fang, Ambuj Tewari, Srijan Sen

Contents

1 Additional Analyses **3**
1.1 Main Effects of Notification Categories on Weekly Outcomes . . . 3
1.2 Comparing Life Insights and Tips . . . 3
1.3 Long-term Effects of Notifications on Mental Health . . . 6
1.4 Additional Moderators . . . 7
1.4.1 Baseline Moderators . . . 8
1.4.2 Main Effects Over Time . . . 8
1.5 Non-Linear Moderation . . . 8
1.6 Summary of Notification Frequencies . . . 11

2 Missing Data and Sensitivity Analyses **12**
2.1 Sensitivity of the Primary Aim Results . . . 13
2.1.1 Dropout Sensitivity . . . 13
2.1.2 Weekly Missingness Sensitivity . . . 13
2.1.3 Conclusions . . . 13
2.2 Sensitivity of Secondary Aim 1 Results . . . 13
2.2.1 Dropout Sensitivity . . . 14
2.2.2 Weekly Missingness Sensitivity . . . 14
2.2.3 Conclusions . . . 14
2.3 Sensitivity of Secondary Aim 2 Results . . . 14
2.3.1 Dropout Sensitivity . . . 14
2.3.2 Weekly Missingness Sensitivity . . . 14
2.3.3 Conclusions . . . 15
2.4 Overall conclusions . . . 15

3 Further Details on the Statistical Methods **16**
3.1 Statistical Model 16
3.1.1 Primary Aim 17
3.1.2 Secondary Aims and Exploratory Sub-aim 17
3.2 Methodology 17
3.3 Implementation 18

1 Additional Analyses

In this section we present additional analyses conducted for the 2018 Intern Health Study MRT.

1.1 Main Effects of Notification Categories on Weekly Outcomes

The first additional analyses conducted were the main effects analyses of all the moderator analyses presented in the main paper. These analyses look at the non-moderated effects of different categories of notifications on various outcomes. The analysis methods are the exact same, except the model no longer contains an interaction between the treatment and moderator (eliminating $b_1 Z_t M_t$). The outcome variables are still aggregated at the weekly-level. These analyses answer the following questions:

1. What is the effect of notifications (of any category) on average daily mood compared to no notifications?

2. What is the effect of mood notifications on average daily mood compared to no notifications?

3. What is the effect of activity notifications on average daily step count compared to no notification?

4. What is the effect of sleep notifications on average daily sleep compared to no notifications?

The results are presented in Table 1. There is strong evidence of a negative effect of notifications on mood. There is weak evidence of a negative effect of mood notifications on mood. There is strong evidence of a positive effect of activity notifications on step counts. Lastly, there is moderate evidence of a positive effect of sleep notifications on sleep. The effect sizes for all of these effects are small.

1.2 Comparing Life Insights and Tips

In addition to comparing notification categories, we were also interested in comparing notification types (life insights or tips). In the 2018 IHS MRT, life-insights

		Outcome		
		**Mood**	**Step**	**Sleep**
Notification Category	General	-0.029 (P = .003)<br>d = -0.020<br>95% CI:<br>-0.048 to -0.010		
	Mood	-0.023 (P = .153)<br>d = -0.016<br>95% CI:<br>-0.054 to 0.009		
	Activity		0.693 (P = .023)<br>d = 0.044<br>95% CI:<br>0.101 to 1.285	
	Sleep			0.051 (P = .073)<br>d = 0.036<br>95% CI:<br>-0.004 to 0.106

Table 1: Effects (p-values) and effect sizes, Cohen's d, 95% confidence intervals of various notification categories on different outcomes. Effects are compared to a baseline of no notification.

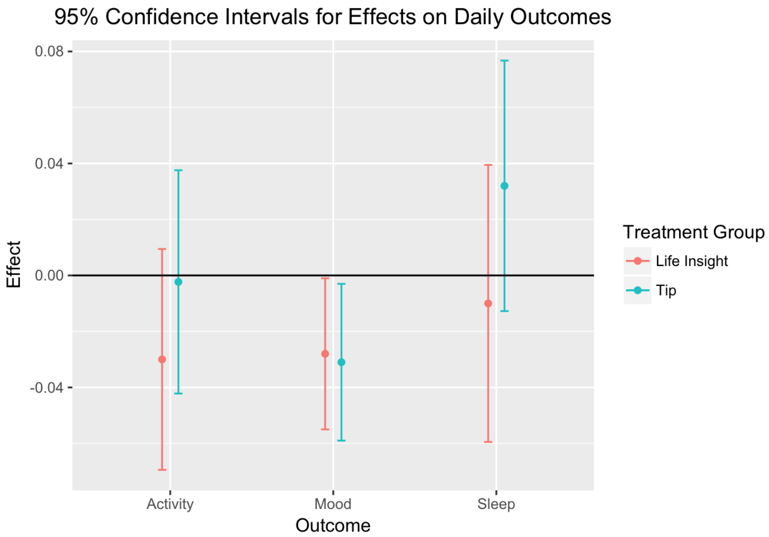

Figure 1: 95% confidence intervals and point estimates of the effects of life insights (compared to no notification) and tips (compared to no notification) on daily step count, mood, and sleep. Activity effects and intervals have been scaled down by 10.

and tips were not randomly assigned, but were instead alternated deterministically. However, the decision between sending a notification and not sending a notification on a given day was randomly assigned (with 50% probability). Hence, for this analysis, the efficacy of different notification types is evaluated by comparing life-insight notification days to no-notification days and comparing tip notification days to no-notification days. Since these randomizations were done at the daily level, the outcomes of interest are also at the daily level. The outcome is daily step count, daily mood, or nightly sleep duration on the day a particular notification type was sent. Again, this analysis uses a weighted and centered least squares estimator. Figure 1 presents 95% confidence intervals and point estimates of these effects.

Figure 1 demonstrates there is moderate evidence that tips perform slightly better than life insights for daily steps and sleep. There is moderate evidence of a positive effect of tips on daily sleep. There is moderate evidence of a negative effect of life insights on daily step count. For daily mood, the effects of both life insights and tips are negative, and there does not appear to be a difference between the two types.

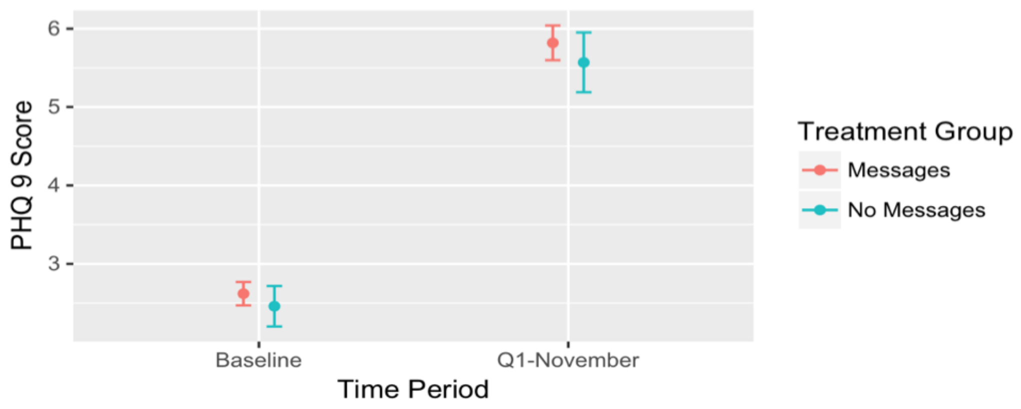

Figure 2: 95% confidence intervals and point estimates of the average PHQ-9 score at baseline and 4 months into internship. Lower score corresponds to a lower frequency of depressive symptoms.

The deterministic alternating between life insights and tips does make causal interpretation of these effects difficult. Nonetheless, the analyses are informative and provide some evidence of the different effects of notification types.

1.3 Long-term Effects of Notifications on Mental Health

We were also interested in the long-term effects of notifications on intern mental health. To assess the long-term effects, we included an additional baseline randomization prior to the start of the internship. For this randomization, 25% of interns are randomized to receive no notifications for the entire internship, while the other 75% would enter the MRT and receive notifications. To assess the long-term mental health of interns, we use the PHQ-9 [1]. The PHQ-9 score of each intern is measured at baseline (prior to internship) and in November (4 months into the internship). In the 2018 IHS, 546 interns were randomized to not receive notifications for the entire internship, while 1,565 interns were randomized to receive notifications during the internship. Point estimates and 95% confidence intervals of the average PHQ-9 score of each group are shown in Figure 2. For PHQ-9, a lower score corresponds to a lower frequency of depressive symptoms.

Figure 2 demonstrates there is no evidence of a positive effect (i.e., lower PHQ-9) of notifications on average PHQ-9 score. In fact, the average PHQ-9 score for the notification group is slightly larger than for the no notification group. This difference is not statistically significant.

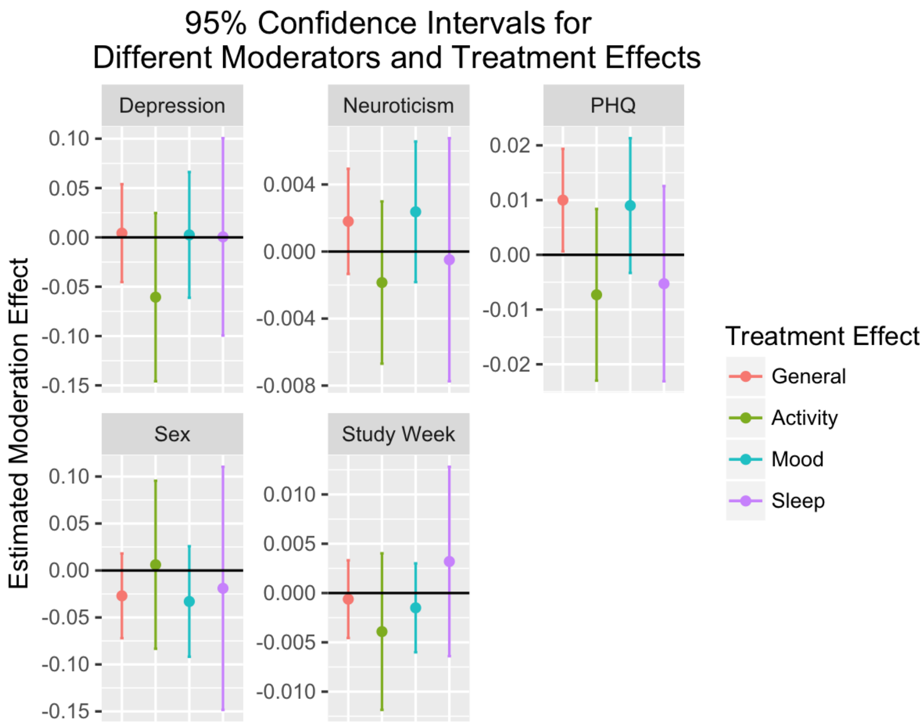

Figure 3: 95% confidence intervals and point estimates of the moderator effects for different moderators and treatment effects. Activity moderation effects and intervals have been scaled down by 10.

1.4 Additional Moderators

In addition to the real-time moderators presented in the paper, we analyzed additional moderators. We were interested in how baseline variables may moderate the main effects in Section 1.1. We were also interested in how the main effects changed over time, which can be assessed by analyzing *time in study* as a moderator. The analysis methods are the exact same as the main paper, except the moderator (M_t) is now defined as either a baseline variable of interest, or time in study. For each moderator of interest, Figure 3 illustrates the 95% confidence intervals and point estimates of the moderation effect (b_1) of every notification effect in Section 1.1.

1.4.1 Baseline Moderators

We assess four potential baseline moderators sex (male = 0, female = 1), baseline PHQ-9 (larger values correspond to more depressive symptoms), baseline neuroticism (larger values correspond to higher levels of neuroticism), baseline depression history (previous history of depression = 1, otherwise = 0). The results can be found in Figure 3. There is some evidence that baseline PHQ-9 positively moderates the effect of general notifications on mood. For other baseline moderators and notification effects, there is not strong evidence of moderation.

1.4.2 Main Effects Over Time

In mobile health studies, due to decreased engagement and app usage, there is evidence of treatment effects deteriorating over time [2]. In this section, we demonstrate how the main effects presented in Section 1.1 changed over time. We included study week ($t = 1, \ldots, 26$) as a moderator. The estimated moderation of time is presented in Figure 3. Though the moderator effect was estimated to be negative for most main effects (implying worse treatment effects later in the study) there is not strong evidence for any of these moderators. Hence, we conclude that there is not strong evidence that any of the four treatment effects vary over time.

1.5 Non-Linear Moderation

The analysis within the main paper used a *linear* model for the moderation. That is, the model for the treatment effect was specified as a linear function of the moderator, namely $b_0 Z_t + b_1 Z_t M_t = (b_0 + b_1 M_t) Z_t$. In this section we explore potential non-linearities in the moderation. The treatment effect is now modeled as $f(M_t) Z_t$, where f is a smooth, potentially non-linear function of M_t. Other parts of the model remain the same.

f is estimated using penalized basis splines [3]. The mgcv package [4] in R estimates f and plots the estimated function, $\hat{f}$. It also provides 95% point-wise confidence bands for $\hat{f}$. This is done for the primary aim (Figure 4) and secondary aims (Figures 5 and 6).

There are a couple differences between the analysis in this section and the analysis done in the main paper. For one, in the main paper, multiple imputation was used to cope with missing data, and Rubin's rules were used to combine coefficient estimates across multiple imputed data sets. In this analysis, since there is no simple way to combine estimated *functions* across multiple data sets, we plot

$\hat{f}$ for each imputed data set and present a representative example to display here. Fortunately, $\hat{f}$ was similar for each imputed data set. Secondly, the weighted and centered least squares estimator [5] and 'sandwich' standard error estimator [6] has not been extended to penalized basis splines. This analysis is exploratory and intended to provide initial evidence of non-linearities. The causal interpretation and robustness of the basis spline and confidence band estimators used in this analysis are not assessed thoroughly.

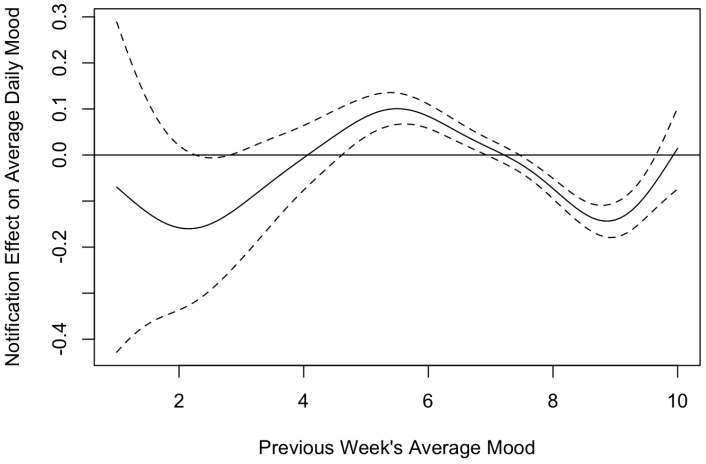

Figure 4: Estimated treatment effects (compared to no notifications) of notifications on average daily mood, at various values of previous week's mood. Dashed lines represent 95% point-wise confidence bands.

Figure 4 shows the primary aim (effect of notifications on mood, moderated by previous mood) with a non-linear moderator. A negative linear moderation is evident when the previous week's average mood score was between 5 and 9. Beyond that range we do notice some evidence of non-linearity. When previous mood scores were above 9, there is evidence that the treatment effect becomes less negative. When previous mood scores were below 5, there is evidence that the treatment effect becomes less positive.

Figure 5 shows secondary aim 1 (effect of activity notifications on steps, moderated by previous steps) with a non-linear moderator. A negative linear moderation is evident when the previous week's average daily step count was below 10,000 steps. When previous step count was above 10,000, there is evidence that the treatment effect does not become negative and instead levels off at 0. This shows that activity notifications may have not *negatively* impacted interns when

their previous step count was high. Rather, notifications may have not affected them at all. Note also that we did not transform the notification effect back into raw step counts (as was done in the paper).

Figure 6 shows secondary aim 2 (effect of sleep notifications on sleep, moderated by previous sleep) with a non-linear moderator. A negative linear moderation is evident when the previous week's average daily sleep hours were below 6. When previous sleep hours were above 6, the treatment effect appears to level off at 0. The effect then dropped off below 0 after previous sleep hours were larger than 8, however the confidence bands are wide. Figure 6 suggests that the negative effect at high previous sleep values may be weaker than suggested by the main paper. Note also that we did not transform the effect back into sleep minutes (as was done in the paper).

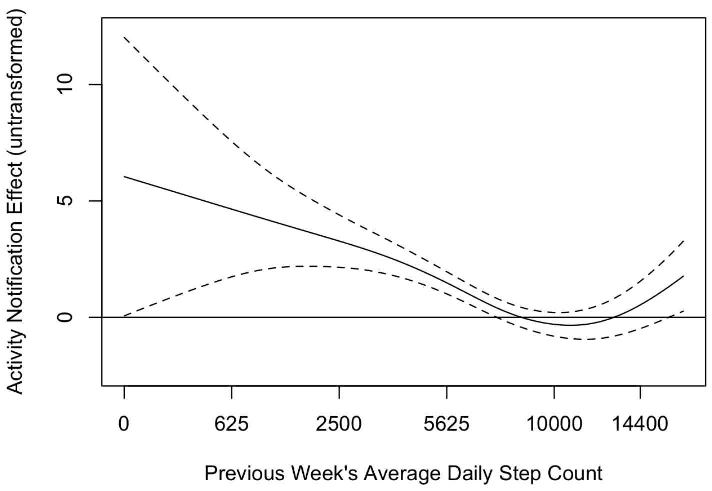

Figure 5: Estimated treatment effects (compared to no notifications) of activity notifications on average daily steps, at various values of previous week's step count. Dashed lines represent 95% point-wise confidence bands.

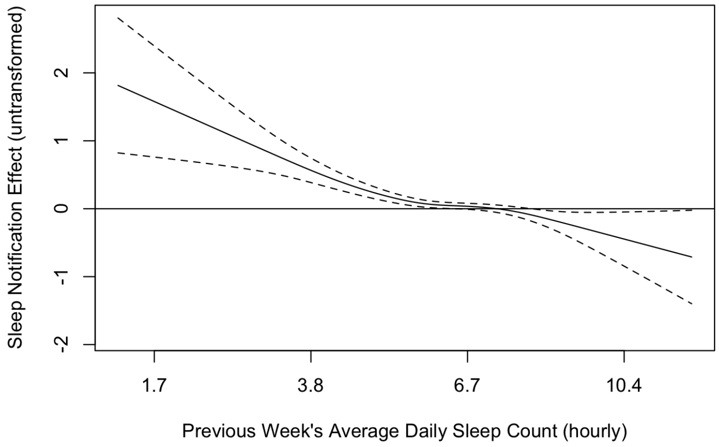

Figure 6: Estimated treatment effects (compared to no notifications) of sleep notifications on average daily sleep, at various values of previous week's sleep hours. Dashed lines represent 95% point-wise confidence bands.

1.6 Summary of Notification Frequencies

On weeks where users receive notifications, there is a 50% chance that the user will receive a notification on any given day. Because of this, there is variability in the number of notifications a user may receive on any given week. In Table 2, we provided the empirical frequencies of how often users receive 0, 1, 2, . . ., or 7 notifications during a notification week in the trial. These frequencies closely resemble the expected frequencies.

Number of notifications	0	1	2	3	4	5	6	7
Frequency	0.8%	5.5%	16.5%	27.1%	27.4%	16.4%	5.6%	0.8%

Table 2: Empirical frequencies for how often users receive 0, 1, 2, . . ., or 7 notifications during a notification week.

2 Missing Data and Sensitivity Analyses

Missing data occurred throughout the intern health study. The primary aim's outcome (mood) was missing because interns failed to self-report. The other two secondary outcomes (sleep and step count) were missing as collection required interns to wear their Fitbits. Figure 7 displays the percentage of interns with at least one non-missing sleep, step, or mood observation for each week in the study. There was a downward trend in percentage of users with non-missing data. It is known that attrition over time is a major issue in mobile health studies [2]. In this section, we explore the sensitivity of the main results in the paper to missingness.

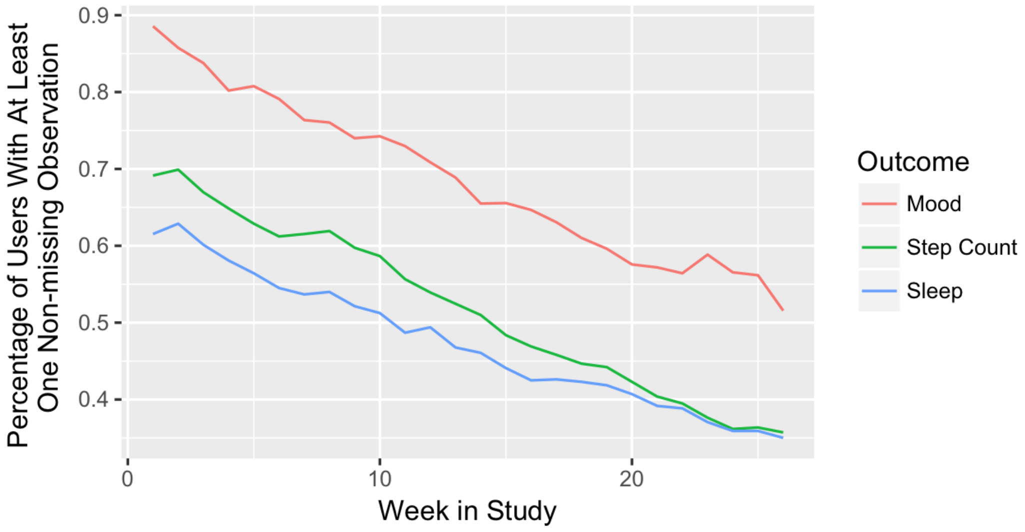

Figure 7: Percentage of interns with at least one non-missing sleep, step, or mood observation for each week in the study

To cope with the missingness, multiple imputation was used in the main paper. Multiple imputation [7] is a robust method for dealing with missing data in which missing data is filled in using predicted values. Another method for handling missing data is complete-case analysis [7], where missing observations are completely dropped from the analysis. Complete-case analysis relies on stronger assumptions than multiple imputation. To assess the sensitivity of our results to the multiple imputation method, the paper's results are compared to complete-case analysis results.

We provided complete-case analyses for two different types of missingness in

the outcome of interest: dropout and weekly missingness. For dropout complete-case analysis, we eliminated imputed data from users who dropped out from the study early. That is, if a user stopped entering mood scores after November 1st, then for the mood outcome analyses, we eliminated that user's imputed data from November 1st onward. For weekly missingness complete-case analysis, we eliminated weeks with a large percentage of missing data in the outcome of interest.

2.1 Sensitivity of the Primary Aim Results

We evaluated the sensitivity of the estimate of the moderation, -0.052 (SE = 0.014, P = .001), of previous week's mood on the effect of notifications (of any category) on average daily mood.

2.1.1 Dropout Sensitivity

We eliminated all imputed data for users after they have dropped out. For example, if a user has stopped entering mood scores after November 1st, we removed all data for that user after November 1st from the analysis.

The new estimate of the moderator is -0.039 (SE = 0.014, P = .006).

2.1.2 Weekly Missingness Sensitivity

In our analysis, we eliminated all weeks where more than 5 daily mood scores are missing.

The new estimate of the moderator is -0.024 (SE = 0.013, P = .076).

2.1.3 Conclusions

The primary aim conclusions are mildly sensitive to missingness. The size of the estimated moderation for the primary aim was reduced when eliminating dropouts or weeks with a large amount of missingness. The sign of the moderation remained negative, matching the conclusions made in the paper.

2.2 Sensitivity of Secondary Aim 1 Results

We evaluated the sensitivity of the estimate of the moderation, -0.039 (SE = 0.015, P = .013), of previous week's step count on the effect of activity notifications on average daily step count.

2.2.1 Dropout Sensitivity

We eliminated all imputed data for users after they have dropped out. For example, if a user has no step count data after November 1st we removed all data for that user after November 1st from the analysis.

The new estimate of the moderator is -0.003 (SE = 0.020, P = .874)

2.2.2 Weekly Missingness Sensitivity

In our analysis, we eliminated all weeks where more than 5 daily step counts are missing.

The new estimate of the moderator is 0.004 (SE = 0.021, P = .858)

2.2.3 Conclusions

The secondary aim 1 conclusions are very sensitive to missingness. For both dropout and weekly missingness, the moderation effect is now very close to 0.

2.3 Sensitivity of Secondary Aim 2 Results

We evaluated the sensitivity of the estimate of the moderation, -0.075 (SE = 0.018, $P < .001$), of previous week's sleep time on the effect of sleep notifications on average daily sleep.

2.3.1 Dropout Sensitivity

We eliminated all imputed data for users after they have dropped out. For example, if a user has no sleep data after November 1st, we removed all data for that user after November 1st from the analysis.

The new estimate of the moderator is -0.034 (SE = 0.025, P = .173)

2.3.2 Weekly Missingness Sensitivity

In our analysis, we eliminated all weeks where more than 5 daily sleep times are missing.

The new estimate of the moderator is -0.044 (SE = 0.022, P = .044)

2.3.3 Conclusions

The secondary aim 2 conclusions are mildly sensitive to missingness. The size of moderation for secondary aim 2 was reduced when eliminating dropouts or weeks with a large amount of missingness. The sign the moderation remained negative, matching the conclusions made in the paper.

2.4 Overall conclusions

Overall, this analysis has demonstrated some sensitivity of the conclusions to missingness in the data. The conclusions of the primary aim and secondary aim 2 seem to be robust to missingness. The conclusions for secondary aim 1, however, are very sensitive.

The reduction in effect size after dropping imputed data from the analysis could indicate a few things. In the worst case, it could indicate that the large effect size is an artifact of the imputation model itself. That is, the methods used to overcome the missing data are biasing the estimates away from 0. On the other hand, the reduction in effect size could indicate that the effect is strongest for interns with a large amount of missingness. In this case, dropping the imputed data would bias the estimates towards 0. One of the challenges of dealing with missing data is not knowing the truth because the data needed to distinguish these two scenarios is missing.

3 Further Details on the Statistical Methods

In this section, we provide further details on the statistical model, methodology, and implementation used in the main paper. In this section, **boldface** is used to indicate multi-dimensional column vectors. $\mathbf{X}'$ indicates the transpose of vector $\mathbf{X}$.

3.1 Statistical Model

In all aims $\mathbf{X_t}$ is an 11-dimensional vector of control covariates. The control covariates are variables associated with the outcome, Y_t, and are measured prior to each weekly randomization. The purpose of the control covariates is to reduce the variation in the outcome and reduce the standard error when estimating the treatment effect of interest. The control covariates used in all analyses are baseline sex, baseline PHQ-9 score, baseline depression history, baseline neuroticism, pre-internship average daily mood, pre-internship average daily square root step count, pre-internship average daily square root sleep minutes, previous week's average daily mood, previous week's average daily square root step count, previous week's average daily square root sleep minutes, and study week. $\boldsymbol{a_0}$ is the corresponding 11-dimensional vector of coefficients for the 11 control covariates. M_t is the 1-dimensional moderator of interest. Y_t is the outcome of interest. Z_t is the treatment indicator. In the primary aim, Z_t is 1-dimensional, with $Z_t = 1$ indicating a notification week of any category and $Z_t = 0$ indicating a no notification week. In the secondary aims and exploratory sub-aim, the treatment is no longer binary since there are 4 possible notification categories. Z_t is now a 3-dimensional vector which encodes 3 indicator variables: activity notification weeks ($\mathbf{Z_t} = (1, 0, 0)'$), sleep notification weeks ($\mathbf{Z_t} = (0, 1, 0)'$), mood notification weeks ($\mathbf{Z_t} = (0, 0, 1)'$), or no-notification weeks ($\mathbf{Z_t} = (0, 0, 0)'$).

A linear model was used as a working model for the moderator analysis. The model is a 'working' model, as indicated by "=", because the estimation methods do not require correct specification of parts of the model not interacted with treatment, such as $\boldsymbol{a_0}'\mathbf{X_t} + a_1 M_t$ below.

For the primary aim, the coefficient (b_0) of Z_t is interpreted as the treatment effect of notifications, compared to no notifications, when the moderator M_t is 0. The coefficient (b_1) for the interaction of Z_t and M_t is interpreted as the change in treatment effect of treatment Z_t on Y_t for a 1 unit change in M_t. It is the moderation effect of interest. The moderation effect, b_1, is an average effect. It is average over time in the study and user-specific variables. Similar interpretations

hold for the non-binary treatment in the secondary and exploratory aims.

Below are the working models used in analyses.

3.1.1 Primary Aim

$E(Y_t|\mathbf{X_t}, M_t, Z_t)$ "=" $\boldsymbol{a_0'}\mathbf{X_t} + a_1 M_t + b_0 Z_t + b_1 Z_t M_t =$
$a_{01}X_{t1} + a_{02}X_{t2} + \ldots + a_{0,11}X_{t11} + a_1 M_t + b_0 Z_t + b_1 Z_t M_t$

In the primary aim, Y_t is average daily mood and M_t is average daily mood of the previous week.

3.1.2 Secondary Aims and Exploratory Sub-aim

$E(Y_t|\mathbf{X_t}, M_t, \mathbf{Z_t})$ "=" $\boldsymbol{a_0'}\mathbf{X_t} + a_1 M_t + \boldsymbol{b_0'}\mathbf{Z_t} + \boldsymbol{b_1'}\mathbf{Z_t} M_t =$
$\boldsymbol{a_0'}\mathbf{X_t} + a_1 M_t + b_{01}Z_{t1} + b_{02}Z_{t2} + b_{03}Z_{t3} + b_{11}Z_{t1}M_t + b_{12}Z_{t2}M_t + b_{13}Z_{t3}M_t$

In secondary aim 1, Y_t is average daily square root step count and M_t is average daily square root step count of the previous week. In secondary aim 2, Y_t is average daily square root sleep count and M_t is average daily square root sleep count of the previous week. In the exploratory aim, Y_t is average daily mood and M_t is average daily mood of the previous week.

3.2 Methodology

To estimate the coefficients of interest, we used the weighted and centered least squares estimator outlined in [5]. The method is robust to misspecification of parts of the model not interacted with treatment.

The methods developed in [5] are useful for robust estimation when treatment assignment probabilities are time-varying (for example, an MRT where the probability of treatment assignment is based on data collected throughout the trial). In the IHS MRT, the treatment assignment probabilities were constant across weeks. Because of this, in the estimating equation, all weights were equal to 1 and the centering term, ρ, was constant ($\rho = 0.75$ in primary aim, $\boldsymbol{\rho} = (0.25, 0.25, 0.25)'$ in secondary/exploratory aims).

The method also uses the standard 'sandwich' estimator for robust standard error estimation [6]. As mentioned in [5], an independent working correlation matrix was used to prevent biased estimation of coefficients.

The estimating equation approach with robust standard error estimation is advantageous because it does not require distributional assumptions on the continu-

ous outcomes. The approach also permits dependencies between observations in the data, as was expected with the repeatedly measured outcomes.

3.3 Implementation

The method was implemented in R using the package geepack [8]. The method was implemented using the standard *geeglm* function with a centered treatment indicator. That is, for the primary aim Z_t was transformed to $Z_t - \rho$, and for the secondary aims and exploratory aims $\mathbf{Z_t}$ was transformed to $\mathbf{Z_t} - \boldsymbol{\rho}$.

Since multiple imputation was used to deal with missingness, the coefficients and standard errors were estimated for each imputed data set. The coefficients and standard errors were combined across multiple imputations using Rubin's rules. The *testestimates* function in the mitml R package [9] was used to combine estimates.

Code will be made available on the first author's website.

References

[1] K. Kroenke, R. L. Spitzer, and J. B. Williams, "The PHQ-9: validity of a brief depression severity measure," *Journal of General Internal Medicine*, vol. 16, no. 9, pp. 606–613, 2001.

[2] G. Eysenbach, "The law of attrition," *Journal of Medical Internet Research*, vol. 7, no. 1, p. e11, 2005.

[3] S. N. Wood and N. H. Augustin, "Gams with integrated model selection using penalized regression splines and applications to environmental modelling," *Ecological modelling*, vol. 157, no. 2-3, pp. 157–177, 2002.

[4] S. Wood. (2012) mgcv. [Online]. Available: https://cran.r-project.org/web/packages/mgcv/mgcv.pdf

[5] A. Boruvka, D. Almirall, K. Witkiewitz, and S. A. Murphy, "Assessing time-varying causal effect moderation in mobile health," *Journal of the American Statistical Association*, vol. 113, no. 523, pp. 1112–1121, 2018.

[6] P. J. Huber *et al.*, "The behavior of maximum likelihood estimates under nonstandard conditions," in *Proceedings of the Fifth Berkeley Symposium on Mathematical Statistics and Probability*, vol. 1, no. 1. University of California Press, 1967, pp. 221–233.

[7] R. J. Little and D. B. Rubin, *Statistical analysis with missing data*. Wiley, 2019, vol. 793.

[8] U. Halekoh, S. Højsgaard, J. Yan *et al.*, "The R package geepack for generalized estimating equations," *Journal of Statistical Software*, vol. 15, no. 2, pp. 1–11, 2006.

[9] S. Grund, A. Robitzsch, and O. Lüdtke, "mitml: Tools for multiple imputation in multilevel modeling," *Retreived from: https://cran.r-project.org/web/packages/mitml/index.html*, 2016.
