# Supplementary material for: Assessing Real-Time Moderation for Developing Adaptive Mobile Health Interventions for Medical Interns: Micro-Randomized Trial
Source: J Med Internet Res. 2020 Mar 31;22(3):e15033. doi: 10.2196/15033 (PMC7157494; doi:10.2196/15033)
Supplement: Multimedia Appendix 2 [file jmir_v22i3e15033_app2.pdf]

CONSORT-EHEALTH (V 1.6.1) - Submission/Publication Form

The CONSORT-EHEALTH checklist is intended for authors of randomized trials evaluating web-based and Internet-based applications/interventions, including mobile interventions, electronic games (incl multiplayer games), social media, certain telehealth applications, and other interactive and/or networked electronic applications. Some of the items (e.g. all subitems under item 5 - description of the intervention) may also be applicable for other study designs.

The goal of the CONSORT EHEALTH checklist and guideline is to be
a) a guide for reporting for authors of RCTs,
b) to form a basis for appraisal of an ehealth trial (in terms of validity)

CONSORT-EHEALTH items/subitems are MANDATORY reporting items for studies published in the Journal of Medical Internet Research and other journals / scientific societies endorsing the checklist.

Items numbered 1., 2., 3., 4a., 4b etc are original CONSORT or CONSORT-NPT (non-pharmacologic treatment) items.
Items with Roman numerals (i., ii, iii, iv etc.) are CONSORT-EHEALTH extensions/clarifications.

As the CONSORT-EHEALTH checklist is still considered in a formative stage, we would ask that you also RATE ON A SCALE OF 1-5 how important/useful you feel each item is FOR THE PURPOSE OF THE CHECKLIST and reporting guideline (optional).

Mandatory reporting items are marked with a red *.
In the textboxes, either copy & paste the relevant sections from your manuscript into this form - please include any quotes from your manuscript in QUOTATION MARKS,
or answer directly by providing additional information not in the manuscript, or elaborating on why the item was not relevant for this study.

YOUR ANSWERS WILL BE PUBLISHED AS A SUPPLEMENTARY FILE TO YOUR PUBLICATION IN JMIR AND ARE CONSIDERED PART OF YOUR PUBLICATION (IF ACCEPTED).
Please fill in these questions diligently. Information will not be copyedited, so please use proper spelling and grammar, use correct capitalization, and avoid abbreviations.

DO NOT FORGET TO SAVE AS PDF _AND_ CLICK THE SUBMIT BUTTON SO YOUR ANSWERS ARE IN OUR DATABASE !!!

Citation Suggestion (if you append the pdf as Appendix we suggest to cite this paper in the caption):
Eysenbach G, CONSORT-EHEALTH Group
CONSORT-EHEALTH: Improving and Standardizing Evaluation Reports of Web-based and Mobile Health Interventions
J Med Internet Res 2011;13(4):e126
URL: http://www.jmir.org/2011/4/e126/
doi: 10.2196/jmir.1923
PMID: 22209829

* Required

Your name *

First Last

Timothy NeCamp

Primary Affiliation (short), City, Country *

University of Toronto, Toronto, Canada

University of Michigan, Ann Arbor, Unite[illegible]

Your e-mail address *

abc@gmail.com

tnecamp@umich.edu

Title of your manuscript *

Provide the (draft) title of your manuscript.

Assessing Real-Time Moderation for Developing Adaptive Mobile Health Interventions for Medical Interns: A Micro-randomized Trial

Name of your App/Software/Intervention *

If there is a short and a long/alternate name, write the short name first and add the long name in brackets.

Intern Health Study mobile app

Evaluated Version (if any)

e.g. "V1", "Release 2017-03-01", "Version 2.0.27913"

Your answer

Language(s) *

What language is the intervention/app in? If multiple languages are available, separate by comma (e.g. "English, French")

English

URL of your Intervention Website or App

e.g. a direct link to the mobile app on app in appstore (itunes, Google Play), or URL of the website. If the intervention is a DVD or hardware, you can also link to an Amazon page.

https://www.srijan-sen-lab.com/intern-h

URL of an image/screenshot (optional)

Your answer

Accessibility *

Can an enduser access the intervention presently?

- ○ access is free and open
- ◉ access only for special usergroups, not open
- ○ access is open to everyone, but requires payment/subscription/in-app purchases
- ○ app/intervention no longer accessible
- ○ Other:

Primary Medical Indication/Disease/Condition *

e.g. "Stress", "Diabetes", or define the target group in brackets after the condition, e.g. "Autism (Parents of children with)", "Alzheimers (Informal Caregivers of)"

Depression, Mood, Sleep, Physical Activ

Primary Outcomes measured in trial *

comma-separated list of primary outcomes reported in the trial

Mood

Secondary/other outcomes

Are there any other outcomes the intervention is expected to affect?

Sleep, Physical Activity

Recommended "Dose" *

What do the instructions for users say on how often the app should be used?

- (selected) Approximately Daily
- Approximately Weekly
- Approximately Monthly
- Approximately Yearly
- "as needed"
- Other:

Approx. Percentage of Users (starters) still using the app as recommended after 3 months *

- ○ unknown / not evaluated
- ○ 0-10%
- ○ 11-20%
- ○ 21-30%
- ○ 31-40%
- ○ 41-50%
- ○ 51-60%
- ○ 61-70%
- ◉ 71%-80%
- ○ 81-90%
- ○ 91-100%
- ○ Other:

Overall, was the app/intervention effective? *

- ( ) yes: all primary outcomes were significantly better in intervention group vs control
- (•) partly: SOME primary outcomes were significantly better in intervention group vs control
- ( ) no statistically significant difference between control and intervention
- ( ) potentially harmful: control was significantly better than intervention in one or more outcomes
- ( ) inconclusive: more research is needed
- ( ) Other:

Article Preparation Status/Stage *

At which stage in your article preparation are you currently (at the time you fill in this form)

- ( ) not submitted yet - in early draft status
- (•) not submitted yet - in late draft status, just before submission
- ( ) submitted to a journal but not reviewed yet
- ( ) submitted to a journal and after receiving initial reviewer comments
- ( ) submitted to a journal and accepted, but not published yet
- ( ) published
- ( ) Other:

Journal *

If you already know where you will submit this paper (or if it is already submitted), please provide the journal name (if it is not JMIR, provide the journal name under "other")

- ○ not submitted yet / unclear where I will submit this
- ◉ Journal of Medical Internet Research (JMIR)
- ○ JMIR mHealth and UHealth
- ○ JMIR Serious Games
- ○ JMIR Mental Health
- ○ JMIR Public Health
- ○ JMIR Formative Research
- ○ Other JMIR sister journal
- ○ Other:

Is this a full powered effectiveness trial or a pilot/feasibility trial? *

- ◉ Pilot/feasibility
- ○ Fully powered

Manuscript tracking number *

If this is a JMIR submission, please provide the manuscript tracking number under "other" (The ms tracking number can be found in the submission acknowledgement email, or when you login as author in JMIR. If the paper is already published in JMIR, then the ms tracking number is the four-digit number at the end of the DOI, to be found at the bottom of each published article in JMIR)

- (x) no ms number (yet) / not (yet) submitted to / published in JMIR
- ( ) Other:

TITLE AND ABSTRACT

1a) TITLE: Identification as a randomized trial in the title

1a) Does your paper address CONSORT item 1a? *

I.e does the title contain the phrase "Randomized Controlled Trial"? (if not, explain the reason under "other")

- ( ) yes
- (x) Other: It contains "Micro-randomized Trial" as this was a micro-randomizec

1a-i) Identify the mode of delivery in the title

Identify the mode of delivery. Preferably use “web-based” and/or “mobile” and/or “electronic game” in the title. Avoid ambiguous terms like “online”, “virtual”, “interactive”. Use “Internet-based” only if Intervention includes non-web-based Internet components (e.g. email), use “computer-based” or “electronic” only if offline products are used. Use “virtual” only in the context of “virtual reality” (3-D worlds). Use “online” only in the context of “online support groups”. Complement or substitute product names with broader terms for the class of products (such as “mobile” or “smart phone” instead of “iphone”), especially if the application runs on different platforms.

	1	2	3	4	5	
subitem not at all important	( )	( )	( )	(x)	( )	essential

Does your paper address subitem 1a-i? *

Copy and paste relevant sections from manuscript title (include quotes in quotation marks "like this" to indicate direct quotes from your manuscript), or elaborate on this item by providing additional information not in the ms, or briefly explain why the item is not applicable/relevant for your study

Our title states "Mobile Health Interventions"

1a-ii) Non-web-based components or important co-interventions in title

Mention non-web-based components or important co-interventions in title, if any (e.g., "with telephone support").

	1	2	3	4	5	
subitem not at all important	○	◉	○	○	○	essential

Does your paper address subitem 1a-ii?

Copy and paste relevant sections from manuscript title (include quotes in quotation marks "like this" to indicate direct quotes from your manuscript), or elaborate on this item by providing additional information not in the ms, or briefly explain why the item is not applicable/relevant for your study

There were none.

1a-iii) Primary condition or target group in the title

Mention primary condition or target group in the title, if any (e.g., "for children with Type I Diabetes")
Example: A Web-based and Mobile Intervention with Telephone Support for Children with Type I Diabetes: Randomized Controlled Trial

	1	2	3	4	5	
subitem not at all important	○	○	○	◉	○	essential

Does your paper address subitem 1a-iii? *

Copy and paste relevant sections from manuscript title (include quotes in quotation marks "like this" to indicate direct quotes from your manuscript), or elaborate on this item by providing additional information not in the ms, or briefly explain why the item is not applicable/relevant for your study

Title says "Medical Interns"

1b) ABSTRACT: Structured summary of trial design, methods, results, and conclusions

NPT extension: Description of experimental treatment, comparator, care providers, centers, and blinding status.

1b-i) Key features/functionalities/components of the intervention and comparator in the METHODS section of the ABSTRACT

Mention key features/functionalities/components of the intervention and comparator in the abstract. If possible, also mention theories and principles used for designing the site. Keep in mind the needs of systematic reviewers and indexers by including important synonyms. (Note: Only report in the abstract what the main paper is reporting. If this information is missing from the main body of text, consider adding it)

	1	2	3	4	5	
subitem not at all important	○	○	○	○	○	essential

Does your paper address subitem 1b-i? *

Copy and paste relevant sections from the manuscript abstract (include quotes in quotation marks "like this" to indicate direct quotes from your manuscript), or elaborate on this item by providing additional information not in the ms, or briefly explain why the item is not applicable/relevant for your study

Yes, "The study investigated when to provide mHealth interventions to individuals in stressful work environments in order to improve their behavior and mental health. The mHealth interventions targeted three categories of behavior: mood, activity, and sleep"

"Every week, interns were randomly assigned to receive push notifications of a particular category (mood, activity, sleep, or no notifications). "

1b-ii) Level of human involvement in the METHODS section of the ABSTRACT

Clarify the level of human involvement in the abstract, e.g., use phrases like “fully automated” vs. “therapist/nurse/care provider/physician-assisted” (mention number and expertise of providers involved, if any). (Note: Only report in the abstract what the main paper is reporting. If this information is missing from the main body of text, consider adding it)

	1	2	3	4	5	
subitem not at all important	(●)	○	○	○	○	essential

Does your paper address subitem 1b-ii?

Copy and paste relevant sections from the manuscript abstract (include quotes in quotation marks "like this" to indicate direct quotes from your manuscript), or elaborate on this item by providing additional information not in the ms, or briefly explain why the item is not applicable/relevant for your study

There was no human involvement.

1b-iii) Open vs. closed, web-based (self-assessment) vs. face-to-face assessments in the METHODS section of the ABSTRACT

Mention how participants were recruited (online vs. offline), e.g., from an open access website or from a clinic or a closed online user group (closed usergroup trial), and clarify if this was a purely web-based trial, or there were face-to-face components (as part of the intervention or for assessment). Clearly say if outcomes were self-assessed through questionnaires (as common in web-based trials). Note: In traditional offline trials, an open trial (open-label trial) is a type of clinical trial in which both the researchers and participants know which treatment is being administered. To avoid confusion, use “blinded” or “unblinded” to indicated the level of blinding instead of “open”, as “open” in web-based trials usually refers to “open access” (i.e. participants can self-enrol). (Note: Only report in the abstract what the main paper is reporting. If this information is missing from the main body of text, consider adding it)

	1	2	3	4	5	
subitem not at all important	◉	○	○	○	○	essential

Does your paper address subitem 1b-iii?

Copy and paste relevant sections from the manuscript abstract (include quotes in quotation marks "like this" to indicate direct quotes from your manuscript), or elaborate on this item by providing additional information not in the ms, or briefly explain why the item is not applicable/relevant for your study

Yes, partially. We didn not mention blinding and recruitment as this was too much for the short abstract.
"The interventions aimed to improve three different outcomes, weekly mood (assessed via a daily survey), weekly step count, and weekly sleep time."
"Every day in the study, we collected interns’ daily mood valence, sleep, and step data. "

1b-iv) RESULTS section in abstract must contain use data

Report number of participants enrolled/assessed in each group, the use/uptake of the intervention (e.g., attrition/adherence metrics, use over time, number of logins etc.), in addition to primary/secondary outcomes. (Note: Only report in the abstract what the main paper is reporting. If this information is missing from the main body of text, consider adding it)

	1	2	3	4	5	
subitem not at all important	○	○	◉	○	○	essential

Does your paper address subitem 1b-iv?

Copy and paste relevant sections from the manuscript abstract (include quotes in quotation marks "like this" to indicate direct quotes from your manuscript), or elaborate on this item by providing additional information not in the ms, or briefly explain why the item is not applicable/relevant for your study

Yes.
"We conducted a 6-month micro-randomized trial (MRT) on 1,565 medical interns."

1b-v) CONCLUSIONS/DISCUSSION in abstract for negative trials

Conclusions/Discussions in abstract for negative trials: Discuss the primary outcome - if the trial is negative (primary outcome not changed), and the intervention was not used, discuss whether negative results are attributable to lack of uptake and discuss reasons. (Note: Only report in the abstract what the main paper is reporting. If this information is missing from the main body of text, consider adding it)

	1	2	3	4	5	
subitem not at all important	◉	○	○	○	○	essential

Does your paper address subitem 1b-v?

Copy and paste relevant sections from the manuscript abstract (include quotes in quotation marks "like this" to indicate direct quotes from your manuscript), or elaborate on this item by providing additional information not in the ms, or briefly explain why the item is not applicable/relevant for your study

No, it was not a negative trial.

INTRODUCTION

2a) In INTRODUCTION: Scientific background and explanation of rationale

2a-i) Problem and the type of system/solution

Describe the problem and the type of system/solution that is object of the study: intended as stand-alone intervention vs. incorporated in broader health care program? Intended for a particular patient population? Goals of the intervention, e.g., being more cost-effective to other interventions, replace or complement other solutions? (Note: Details about the intervention are provided in “Methods” under 5)

	1	2	3	4	5	
subitem not at all important	○	○	○	○	○	essential

Does your paper address subitem 2a-i? *

Copy and paste relevant sections from the manuscript (include quotes in quotation marks "like this" to indicate direct quotes from your manuscript), or elaborate on this item by providing additional information not in the ms, or briefly explain why the item is not applicable/relevant for your study

"We assessed time-varying moderators of mobile health interventions targeting three categories: mood, activity, and sleep. Stressful work environments can lead to sleep deprivation and physical inactivity, two behaviors directly associated with depression. To prevent depression among individuals experiencing high stress, it is critical to develop high-quality interventions which can help them maintain and improve their mood, either through targeting mood directly, or by indirectly improving activity and sleep "

2a-ii) Scientific background, rationale: What is known about the (type of) system

Scientific background, rationale: What is known about the (type of) system that is the object of the study (be sure to discuss the use of similar systems for other conditions/diagnoses, if appropiate), motivation for the study, i.e. what are the reasons for and what is the context for this specific study, from which stakeholder viewpoint is the study performed, potential impact of findings [2]. Briefly justify the choice of the comparator.

	1	2	3	4	5	
subitem not at all important	○	○	○	○	○	essential

Does your paper address subitem 2a-ii? *

Copy and paste relevant sections from the manuscript (include quotes in quotation marks "like this" to indicate direct quotes from your manuscript), or elaborate on this item by providing additional information not in the ms, or briefly explain why the item is not applicable/relevant for your study

"Current behavioral theories lack the granularity and adaptivity necessary to inform the timing of the delivery of mobile health interventions. Many theoretical models are non-dynamic—they only consider treatment adaptation based on baseline characteristics, such as sex and depression history. Timing and adapting treatments based on real-time variables is essential for developing high-quality JITAIs"

2b) In INTRODUCTION: Specific objectives or hypotheses

Does your paper address CONSORT subitem 2b? *

Copy and paste relevant sections from the manuscript (include quotes in quotation marks "like this" to indicate direct quotes from your manuscript), or elaborate on this item by providing additional information not in the ms, or briefly explain why the item is not applicable/relevant for your study

Yes. The aims are mentioned in the sections titled Primary Aim and Secondary Aims

METHODS

3a) Description of trial design (such as parallel, factorial) including allocation ratio

Does your paper address CONSORT subitem 3a? *

Copy and paste relevant sections from the manuscript (include quotes in quotation marks "like this" to indicate direct quotes from your manuscript), or elaborate on this item by providing additional information not in the ms, or briefly explain why the item is not applicable/relevant for your study

Yes, it is described as a micro-randomized trial and treatment assignment probabilities are provides.

3b) Important changes to methods after trial commencement (such as eligibility criteria), with reasons

Does your paper address CONSORT subitem 3b? *

Copy and paste relevant sections from the manuscript (include quotes in quotation marks "like this" to indicate direct quotes from your manuscript), or elaborate on this item by providing additional information not in the ms, or briefly explain why the item is not applicable/relevant for your study

There were no method changes after commencement.

3b-i) Bug fixes, Downtimes, Content Changes

Bug fixes, Downtimes, Content Changes: ehealth systems are often dynamic systems. A description of changes to methods therefore also includes important changes made on the intervention or comparator during the trial (e.g., major bug fixes or changes in the functionality or content) (5-iii) and other “unexpected events” that may have influenced study design such as staff changes, system failures/downtimes, etc. [2].

	1	2	3	4	5	
subitem not at all important	○	○	○	◉	○	essential

Does your paper address subitem 3b-i?

Copy and paste relevant sections from the manuscript (include quotes in quotation marks "like this" to indicate direct quotes from your manuscript), or elaborate on this item by providing additional information not in the ms, or briefly explain why the item is not applicable/relevant for your study

There were no bug fixes, downtimes, or content changes after commencement.

4a) Eligibility criteria for participants

Does your paper address CONSORT subitem 4a? *

Copy and paste relevant sections from the manuscript (include quotes in quotation marks "like this" to indicate direct quotes from your manuscript), or elaborate on this item by providing additional information not in the ms, or briefly explain why the item is not applicable/relevant for your study

Yes, under the Participants subsection. "Medical doctors starting their year-long internship in summer of 2018 were eligible to participate in the study. Interns were on-boarded before the start of their internship (between April 2018-June 2018), in which they were instructed to download the study app, were provided Fitbits, completed a baseline survey, and were able to begin entering mood scores. "

4a-i) Computer / Internet literacy

Computer / Internet literacy is often an implicit “de facto” eligibility criterion - this should be explicitly clarified.

	1	2	3	4	5	
subitem not at all important	○	○	○	○	○	essential

Does your paper address subitem 4a-i?

Copy and paste relevant sections from the manuscript (include quotes in quotation marks "like this" to indicate direct quotes from your manuscript), or elaborate on this item by providing additional information not in the ms, or briefly explain why the item is not applicable/relevant for your study

This was not an issue for our study. Participants did need iPhones. This is described under recruitment. "The study app and study participation were restricted to interns using an iPhone, the phone brand used by a majority of interns. "

4a-ii) Open vs. closed, web-based vs. face-to-face assessments:

Open vs. closed, web-based vs. face-to-face assessments: Mention how participants were recruited (online vs. offline), e.g., from an open access website or from a clinic, and clarify if this was a purely web-based trial, or there were face-to-face components (as part of the intervention or for assessment), i.e., to what degree got the study team to know the participant. In online-only trials, clarify if participants were quasi-anonymous and whether having multiple identities was possible or whether technical or logistical measures (e.g., cookies, email confirmation, phone calls) were used to detect/prevent these.

	1	2	3	4	5	
subitem not at all important	○	○	○	○	○	essential

Does your paper address subitem 4a-ii? *

Copy and paste relevant sections from the manuscript (include quotes in quotation marks "like this" to indicate direct quotes from your manuscript), or elaborate on this item by providing additional information not in the ms, or briefly explain why the item is not applicable/relevant for your study

Yes, mentioned under recruitment
"Participants were recruited through emails, which were sent to future interns from 47 different recruitment institutions between April 1, 2018 and June 25, 2018. The recruitment institutions comprised both medical schools, where emails were sent to all graduates, and residency locations, where emails were sent to all incoming interns. 5,233 future interns received the initial email inviting them to participate in the study. 2,134 (41%) interns downloaded the study app, completed the consent form, and filled out the baseline survey sometime before June 25, 2018. The study app and study participation were restricted to interns using an iPhone, the phone brand used by a majority of interns. The 2,134 interns received a Fitbit Charge 2. Of the 2,134, 1,565 interns (73%) were randomly selected to participate in the MRT (see Supplement Section 1 for an explanation of this initial randomization). These 1,565 interns were randomized according to Figure 2. Interns were incentivized to participate in the study by receiving the Fitbit wearable and up to $125, distributed five times throughout the year ($25 each time) based on continued participation."

4a-iii) Information giving during recruitment

Information given during recruitment. Specify how participants were briefed for recruitment and in the informed consent procedures (e.g., publish the informed consent documentation as appendix, see also item X26), as this information may have an effect on user self-selection, user expectation and may also bias results.

	1	2	3	4	5	
subitem not at all important	○	○	○	○	○	essential

Does your paper address subitem 4a-iii?

Copy and paste relevant sections from the manuscript (include quotes in quotation marks "like this" to indicate direct quotes from your manuscript), or elaborate on this item by providing additional information not in the ms, or briefly explain why the item is not applicable/relevant for your study

Yes, mentioned under recruitment
"Participants were recruited through emails, which were sent to future interns from 47 different recruitment institutions between April 1, 2018 and June 25, 2018. The recruitment institutions comprised both medical schools, where emails were sent to all graduates, and residency locations, where emails were sent to all incoming interns. 5,233 future interns received the initial email inviting them to participate in the study. 2,134 (41%) interns downloaded the study app, completed the consent form, and filled out the baseline survey sometime before June 25, 2018. The study app and study participation were restricted to interns using an iPhone, the phone brand used by a majority of interns. The 2,134 interns received a Fitbit Charge 2. Of the 2,134, 1,565 interns (73%) were randomly selected to participate in the MRT (see Supplement Section 1 for an explanation of this initial randomization). These 1,565 interns were randomized according to Figure 2. Interns were incentivized to participate in the study by receiving the Fitbit wearable and up to $125, distributed five times throughout the year ($25 each time) based on continued participation."

4b) Settings and locations where the data were collected

Does your paper address CONSORT subitem 4b? *

Copy and paste relevant sections from the manuscript (include quotes in quotation marks "like this" to indicate direct quotes from your manuscript), or elaborate on this item by providing additional information not in the ms, or briefly explain why the item is not applicable/relevant for your study

Yes, here "Since the primary aim of the study is focused on understanding the effects of interventions on intern mental health, we employ a daily EMA to measure mood valence (see Figure 1-ii). Daily mood is one of two cardinal symptoms of depression [24]. This daily mood EMA is used widely to track mood in depressed patients [25]. There are more widely used measurements of mental health other than mood valence (such as the Patient Health Questionnaire-9, PHQ-9 [26]). However, these questionnaires are more time intensive and their assessment may cause higher non-response rates. Participants are prompted to enter their daily mood every day at a user-specified time between 5 pm and 10 pm.

In addition to collecting EMA data, the app aggregates and displays visual summaries of interns' historical data, including step and sleep counts (collected through the Fitbit) and mood EMA data"

4b-i) Report if outcomes were (self-)assessed through online questionnaires

Clearly report if outcomes were (self-)assessed through online questionnaires (as common in web-based trials) or otherwise.

	1	2	3	4	5	
subitem not at all important	○	○	○	○	○	essential

Does your paper address subitem 4b-i? *

Copy and paste relevant sections from the manuscript (include quotes in quotation marks "like this" to indicate direct quotes from your manuscript), or elaborate on this item by providing additional information not in the ms, or briefly explain why the item is not applicable/relevant for your study

Yes, here
"Since the primary aim of the study is focused on understanding the effects of interventions on intern mental health, we employ a daily EMA to measure mood valence (see Figure 1-ii). Daily mood is one of two cardinal symptoms of depression [24]. This daily mood EMA is used widely to track mood in depressed patients [25]. There are more widely used measurements of mental health other than mood valence (such as the Patient Health Questionnaire-9, PHQ-9 [26]). However, these questionnaires are more time intensive and their assessment may cause higher non-response rates. Participants are prompted to enter their daily mood every day at a user-specified time between 5 pm and 10 pm.

In addition to collecting EMA data, the app aggregates and displays visual summaries of interns' historical data, including step and sleep counts (collected through the Fitbit) and mood EMA data"

4b-ii) Report how institutional affiliations are displayed

Report how institutional affiliations are displayed to potential participants [on ehealth media], as affiliations with prestigious hospitals or universities may affect volunteer rates, use, and reactions with regards to an intervention.(Not a required item – describe only if this may bias results)

	1	2	3	4	5	
subitem not at all important	○	○	○	○	○	essential

Does your paper address subitem 4b-ii?

Copy and paste relevant sections from the manuscript (include quotes in quotation marks "like this" to indicate direct quotes from your manuscript), or elaborate on this item by providing additional information not in the ms, or briefly explain why the item is not applicable/relevant for your study

No

5) The interventions for each group with sufficient details to allow replication, including how and when they were actually administered

5-i) Mention names, credential, affiliations of the developers, sponsors, and owners

Mention names, credential, affiliations of the developers, sponsors, and owners [6] (if authors/evaluators are owners or developer of the software, this needs to be declared in a “Conflict of interest” section or mentioned elsewhere in the manuscript).

	1	2	3	4	5	
subitem not at all important	○	○	○	○	○	essential

Does your paper address subitem 5-i?

Copy and paste relevant sections from the manuscript (include quotes in quotation marks "like this" to indicate direct quotes from your manuscript), or elaborate on this item by providing additional information not in the ms, or briefly explain why the item is not applicable/relevant for your study

No, his was not an issue for us.

5-ii) Describe the history/development process

Describe the history/development process of the application and previous formative evaluations (e.g., focus groups, usability testing), as these will have an impact on adoption/use rates and help with interpreting results.

	1	2	3	4	5	
subitem not at all important	○	○	○	○	○	essential

Does your paper address subitem 5-ii?

Copy and paste relevant sections from the manuscript (include quotes in quotation marks "like this" to indicate direct quotes from your manuscript), or elaborate on this item by providing additional information not in the ms, or briefly explain why the item is not applicable/relevant for your study

No, this was not done as the development process was not unique.

5-iii) Revisions and updating

Revisions and updating. Clearly mention the date and/or version number of the application/intervention (and comparator, if applicable) evaluated, or describe whether the intervention underwent major changes during the evaluation process, or whether the development and/or content was “frozen” during the trial. Describe dynamic components such as news feeds or changing content which may have an impact on the replicability of the intervention (for unexpected events see item 3b).

	1	2	3	4	5	
subitem not at all important	○	○	○	○	○	essential

Does your paper address subitem 5-iii?

Copy and paste relevant sections from the manuscript (include quotes in quotation marks "like this" to indicate direct quotes from your manuscript), or elaborate on this item by providing additional information not in the ms, or briefly explain why the item is not applicable/relevant for your study

No, there was no revising or updating once the trial began.

5-iv) Quality assurance methods

Provide information on quality assurance methods to ensure accuracy and quality of information provided [1], if applicable.

	1	2	3	4	5	
subitem not at all important	○	○	○	○	○	essential

Does your paper address subitem 5-iv?

Copy and paste relevant sections from the manuscript (include quotes in quotation marks "like this" to indicate direct quotes from your manuscript), or elaborate on this item by providing additional information not in the ms, or briefly explain why the item is not applicable/relevant for your study

This was not applicable.

5-v) Ensure replicability by publishing the source code, and/or providing screenshots/screen-capture video, and/or providing flowcharts of the algorithms used

Ensure replicability by publishing the source code, and/or providing screenshots/screen-capture video, and/or providing flowcharts of the algorithms used. Replicability (i.e., other researchers should in principle be able to replicate the study) is a hallmark of scientific reporting.

	1	2	3	4	5	
subitem not at all important	○	○	○	○	○	essential

Does your paper address subitem 5-v?

Copy and paste relevant sections from the manuscript (include quotes in quotation marks "like this" to indicate direct quotes from your manuscript), or elaborate on this item by providing additional information not in the ms, or briefly explain why the item is not applicable/relevant for your study

Link to the code for the analysis is provided on the first author's website. This is mentioned in the paper.

5-vi) Digital preservation

Digital preservation: Provide the URL of the application, but as the intervention is likely to change or disappear over the course of the years; also make sure the intervention is archived (Internet Archive, webcitation.org, and/or publishing the source code or screenshots/videos alongside the article). As pages behind login screens cannot be archived, consider creating demo pages which are accessible without login.

	1	2	3	4	5	
subitem not at all important	○	○	○	○	○	essential

Does your paper address subitem 5-vi?

Copy and paste relevant sections from the manuscript (include quotes in quotation marks "like this" to indicate direct quotes from your manuscript), or elaborate on this item by providing additional information not in the ms, or briefly explain why the item is not applicable/relevant for your study

There is not url to the application. Links to the website where the app info can be found are given in the paper.

5-vii) Access

Access: Describe how participants accessed the application, in what setting/context, if they had to pay (or were paid) or not, whether they had to be a member of specific group. If known, describe how participants obtained "access to the platform and Internet" [1]. To ensure access for editors/reviewers/readers, consider to provide a "backdoor" login account or demo mode for reviewers/readers to explore the application (also important for archiving purposes, see vi).

	1	2	3	4	5	
subitem not at all important	○	○	○	○	○	essential

Does your paper address subitem 5-vii? *

Copy and paste relevant sections from the manuscript (include quotes in quotation marks "like this" to indicate direct quotes from your manuscript), or elaborate on this item by providing additional information not in the ms, or briefly explain why the item is not applicable/relevant for your study

Yes here:
"Participants were recruited through emails, which were sent to future interns from 47 different recruitment institutions between April 1, 2018 and June 25, 2018. The recruitment institutions comprised both medical schools, where emails were sent to all graduates, and residency locations, where emails were sent to all incoming interns. 5,233 future interns received the initial email inviting them to participate in the study. 2,134 (41%) interns downloaded the study app, completed the consent form, and filled out the baseline survey sometime before June 25, 2018. The study app and study participation were restricted to interns using an iPhone, the phone brand used by a majority of interns. The 2,134 interns received a Fitbit Charge 2. Of the 2,134, 1,565 interns (73%) were randomly selected to participate in the MRT (see Supplement Section 1 for an explanation of this initial randomization). These 1,565 interns were randomized according to Figure 2. Interns were incentivized to participate in the study by receiving the Fitbit wearable and up to $125, distributed five times throughout the year ($25 each time) based on continued participation.
"

5-viii) Mode of delivery, features/functionalities/components of the intervention and comparator, and the theoretical framework

Describe mode of delivery, features/functionalities/components of the intervention and comparator, and the theoretical framework [6] used to design them (instructional strategy [1], behaviour change techniques, persuasive features, etc., see e.g., [7, 8] for terminology). This includes an in-depth description of the content (including where it is coming from and who developed it) [1]," whether [and how] it is tailored to individual circumstances and allows users to track their progress and receive feedback" [6]. This also includes a description of communication delivery channels and – if computer-mediated communication is a component – whether communication was synchronous or asynchronous [6]. It also includes information on presentation strategies [1], including page design principles, average amount of text on pages, presence of hyperlinks to other resources, etc. [1].

	1	2	3	4	5	
subitem not at all important	○	○	○	○	○	essential

Does your paper address subitem 5-viii? *

Copy and paste relevant sections from the manuscript (include quotes in quotation marks "like this" to indicate direct quotes from your manuscript), or elaborate on this item by providing additional information not in the ms, or briefly explain why the item is not applicable/relevant for your study

Yes.
"Study participants were provided a Fitbit Charge 2 [22] to collect sleep and activity data, and a phone app downloaded to the intern's phone. The app is able to conduct ecological momentary assessments (EMAs) [23], aggregate and visualize data, and deliver push notifications.

Since the primary aim of the study is focused on understanding the effects of interventions on intern mental health, we employ a daily EMA to measure mood valence (see Figure 1-ii). Daily mood is one of two cardinal symptoms of depression [24]. This daily mood EMA is used widely to track mood in depressed patients [25]. There are more widely used measurements of mental health other than mood valence (such as the Patient Health Questionnaire-9, PHQ-9 [26]). However, these questionnaires are more time intensive and their assessment may cause higher non-response rates. Participants are prompted to enter their daily mood every day at a user-specified time between 5 pm and 10 pm.

In addition to collecting EMA data, the app aggregates and displays visual summaries of interns' historical data, including step and sleep counts (collected through the Fitbit) and mood EMA data. See Figure 1-i. Displaying historical

trends to the intern helps them self-monitor their mood, activity, and sleep trajectories, and could potentially lead to positive reactive behavior change [27]. These displays are a type of ‘pull’ intervention, i.e., interventions that are available at all times but only accessed upon user request. The ‘pull’ component was available to all participants, and assessing its effects was not the focus of this study.

The IHS app also delivers ‘push’ interventions, i.e., interventions delivered without user prompting. Evaluating the push notification intervention is the focus of this study.
"

"Push notifications were provided to the interns through the app, with the goal of improving healthy behavior in a target category of interest: mood, activity, and sleep (i.e., mood notifications improve mood, activity notifications increase physical activity, sleep notifications increase sleep duration).

For all categories, there are two notification types: tips and life insights. Consistent with motivational interviewing approaches [28]–[30], tips are non-data-based notifications that provide autonomy support (e.g., why change) and tools (e.g., how to change) to promote healthy mood, activity, or sleep. Next, consistent with theory [31], [32] and research showing interventions that enhance self-monitoring promote behavior change [33], life insight notifications summarize an individual’s data, to provide reminders and/or reduce the burden of accessing the app to view visualizations."

5-ix) Describe use parameters

Describe use parameters (e.g., intended “doses” and optimal timing for use). Clarify what instructions or recommendations were given to the user, e.g., regarding timing, frequency, heaviness of use, if any, or was the intervention used ad libitum.

	1	2	3	4	5	
subitem not at all important	○	○	○	○	○	essential

Does your paper address subitem 5-ix?

Copy and paste relevant sections from the manuscript (include quotes in quotation marks "like this" to indicate direct quotes from your manuscript), or elaborate on this item by providing additional information not in the ms, or briefly explain why the item is not applicable/relevant for your study

The goal of the study was to discover timing, so optimal timing and doses were not known prior.

5-x) Clarify the level of human involvement

Clarify the level of human involvement (care providers or health professionals, also technical assistance) in the e-intervention or as co-intervention (detail number and expertise of professionals involved, if any, as well as “type of assistance offered, the timing and frequency of the support, how it is initiated, and the medium by which the assistance is delivered”. It may be necessary to distinguish between the level of human involvement required for the trial, and the level of human involvement required for a routine application outside of a RCT setting (discuss under item 21 – generalizability).

	1	2	3	4	5	
subitem not at all important	○	○	○	○	○	essential

Does your paper address subitem 5-x?

Copy and paste relevant sections from the manuscript (include quotes in quotation marks "like this" to indicate direct quotes from your manuscript), or elaborate on this item by providing additional information not in the ms, or briefly explain why the item is not applicable/relevant for your study

No, the human involvement was minimal.

5-xi) Report any prompts/reminders used

Report any prompts/reminders used: Clarify if there were prompts (letters, emails, phone calls, SMS) to use the application, what triggered them, frequency etc. It may be necessary to distinguish between the level of prompts/reminders required for the trial, and the level of prompts/reminders for a routine application outside of a RCT setting (discuss under item 21 – generalizability).

	1	2	3	4	5	
subitem not at all important	○	○	○	○	○	essential

Does your paper address subitem 5-xi? *

Copy and paste relevant sections from the manuscript (include quotes in quotation marks "like this" to indicate direct quotes from your manuscript), or elaborate on this item by providing additional information not in the ms, or briefly explain why the item is not applicable/relevant for your study

Not applicable.

5-xii) Describe any co-interventions (incl. training/support)

Describe any co-interventions (incl. training/support): Clearly state any interventions that are provided in addition to the targeted eHealth intervention, as ehealth intervention may not be designed as stand-alone intervention. This includes training sessions and support [1]. It may be necessary to distinguish between the level of training required for the trial, and the level of training for a routine application outside of a RCT setting (discuss under item 21 – generalizability.

	1	2	3	4	5	
subitem not at all important	○	○	○	○	○	essential

Does your paper address subitem 5-xii? *

Copy and paste relevant sections from the manuscript (include quotes in quotation marks "like this" to indicate direct quotes from your manuscript), or elaborate on this item by providing additional information not in the ms, or briefly explain why the item is not applicable/relevant for your study

Yes, done here "interventions on intern mental health, we employ a daily EMA to measure mood valence (see Figure 1-ii). Daily mood is one of two cardinal symptoms of depression [24]. This daily mood EMA is used widely to track mood in depressed patients [25]. There are more widely used measurements of mental health other than mood valence (such as the Patient Health Questionnaire-9, PHQ-9 [26]). However, these questionnaires are more time intensive and their assessment may cause higher non-response rates. Participants are prompted to enter their daily mood every day at a user-specified time between 5 pm and 10 pm.

In addition to collecting EMA data, the app aggregates and displays visual summaries of interns' historical data, including step and sleep counts (collected through the Fitbit) and mood EMA data. See Figure 1-i. Displaying historical trends to the intern helps them self-monitor their mood, activity, and sleep trajectories, and could potentially lead to positive reactive behavior change [27]. These displays are a type of 'pull' intervention, i.e., interventions that are available at all times but only accessed upon user request. The 'pull' component was available to all participants, and assessing its effects was not the focus of this study.
"

6a) Completely defined pre-specified primary and secondary outcome measures, including how and when they were assessed

Does your paper address CONSORT subitem 6a? *

Copy and paste relevant sections from the manuscript (include quotes in quotation marks "like this" to indicate direct quotes from your manuscript), or elaborate on this item by providing additional information not in the ms, or briefly explain why the item is not applicable/relevant for your study

Yes, this was done under the primary and secondary outcome sections.

6a-i) Online questionnaires: describe if they were validated for online use and apply CHERRIES items to describe how the questionnaires were designed/deployed

If outcomes were obtained through online questionnaires, describe if they were validated for online use and apply CHERRIES items to describe how the questionnaires were designed/deployed [9].

	1	2	3	4	5	
subitem not at all important	○	○	○	○	○	essential

Does your paper address subitem 6a-i?

Copy and paste relevant sections from manuscript text

There were no online questionnaires.

6a-ii) Describe whether and how “use” (including intensity of use/dosage) was defined/measured/monitored

Describe whether and how “use” (including intensity of use/dosage) was defined/measured/monitored (logins, logfile analysis, etc.). Use/adoption metrics are important process outcomes that should be reported in any ehealth trial.

	1	2	3	4	5	
subitem not at all important	○	○	○	○	○	essential

Does your paper address subitem 6a-ii?

Copy and paste relevant sections from manuscript text

We did not have use as a primary outcome. Missingess of EMAs was reported.

6a-iii) Describe whether, how, and when qualitative feedback from participants was obtained

Describe whether, how, and when qualitative feedback from participants was obtained (e.g., through emails, feedback forms, interviews, focus groups).

	1	2	3	4	5	
subitem not at all important	○	○	○	○	○	essential

Does your paper address subitem 6a-iii?

Copy and paste relevant sections from manuscript text

Not applicable.

6b) Any changes to trial outcomes after the trial commenced, with reasons

Does your paper address CONSORT subitem 6b? *

Copy and paste relevant sections from the manuscript (include quotes in quotation marks "like this" to indicate direct quotes from your manuscript), or elaborate on this item by providing additional information not in the ms, or briefly explain why the item is not applicable/relevant for your study

Not applicable.

7a) How sample size was determined

NPT: When applicable, details of whether and how the clustering by care provides or centers was addressed

7a-i) Describe whether and how expected attrition was taken into account when calculating the sample size

Describe whether and how expected attrition was taken into account when calculating the sample size.

	1	2	3	4	5	
subitem not at all important	○	○	○	○	○	essential

Does your paper address subitem 7a-i?

Copy and paste relevant sections from manuscript title (include quotes in quotation marks "like this" to indicate direct quotes from your manuscript), or elaborate on this item by providing additional information not in the ms, or briefly explain why the item is not applicable/relevant for your study

Not applicable. We aimed to recruit as many interns as possible for this exploratory trial.

7b) When applicable, explanation of any interim analyses and stopping guidelines

Does your paper address CONSORT subitem 7b? *

Copy and paste relevant sections from the manuscript (include quotes in quotation marks "like this" to indicate direct quotes from your manuscript), or elaborate on this item by providing additional information not in the ms, or briefly explain why the item is not applicable/relevant for your study

Not applicable.

8a) Method used to generate the random allocation sequence

NPT: When applicable, how care providers were allocated to each trial group

Does your paper address CONSORT subitem 8a? *

Copy and paste relevant sections from the manuscript (include quotes in quotation marks "like this" to indicate direct quotes from your manuscript), or elaborate on this item by providing additional information not in the ms, or briefly explain why the item is not applicable/relevant for your study

This was done automatically with the app.

8b) Type of randomisation; details of any restriction (such as blocking and block size)

Does your paper address CONSORT subitem 8b? *

Copy and paste relevant sections from the manuscript (include quotes in quotation marks "like this" to indicate direct quotes from your manuscript), or elaborate on this item by providing additional information not in the ms, or briefly explain why the item is not applicable/relevant for your study

The randomization is described thoroughly. There is no blocking.

9) Mechanism used to implement the random allocation sequence (such as sequentially numbered containers), describing any steps taken to conceal the sequence until interventions were assigned

Does your paper address CONSORT subitem 9? *

Copy and paste relevant sections from the manuscript (include quotes in quotation marks "like this" to indicate direct quotes from your manuscript), or elaborate on this item by providing additional information not in the ms, or briefly explain why the item is not applicable/relevant for your study

"In order to determine the best time to deliver notifications of different categories, we ran an MRT. The MRT design is pictured in Figure 2. The MRT design and protocol were approved by University of Michigan IRB (UM IRB Protocol #HUM00033029).

The main randomization was the weekly randomization to a specific notification category (mood, activity, sleep) or to no-notification. Thus, we were able to compare how a week of a certain notification category changed intern behavior when compared to a week of no notifications.

The randomization—and the ensuing analysis of effects—occurred at the weekly-level for two reasons. For one, the notifications are not intended to change the interns' behavior in the next few hours, but over the next few days. Randomizing and analyzing effects at the weekly-level, as opposed to daily- or minute-level, permitted discovery of longer-term effects. Secondly, as interns are quite busy, they may not have significant behavior change after receiving a single notification. Instead interns received several notifications of the same category and had a consistent reminder about improving that category.

Given a week when a user was randomized to receiving notifications, every day they were further randomized (with 50% probability) to receive a notification. Hence, for a mood notification week the user received, on average, 3.5 mood notifications that week. The purpose of this randomization is to balance delivering enough notifications to be noticeable and cause behavior change, but not too often that it leads to treatment fatigue [9]. Treatment fatigue is pervasive in mHealth [7] and for individuals with heavy workloads [9].

Another way to prevent treatment fatigue is through increased variability in notifications and the order they are received [34]. For each notification category, the notifications alternated between life insights and tips. Also, given a type and category, each notification was drawn randomly, without replacement, from a corresponding bucket of notifications. The bucket refilled once it was completely emptied. Alternating between life insights and tips increased the day to day variability of the notification framing. Drawing notifications without replacement ensured that users are not receiving repeats of the same notification. Under this scheme, on average, a user did not receive a repeated notification for 16 weeks."

10) Who generated the random allocation sequence, who enrolled participants, and who assigned participants to interventions

Does your paper address CONSORT subitem 10? *

Copy and paste relevant sections from the manuscript (include quotes in quotation marks "like this" to indicate direct quotes from your manuscript), or elaborate on this item by providing additional information not in the ms, or briefly explain why the item is not applicable/relevant for your study

This was done through the Sen Lab, which is mentioned in the paper.

11a) If done, who was blinded after assignment to interventions (for example, participants, care providers, those assessing outcomes) and how

NPT: Whether or not administering co-interventions were blinded to group assignment

11a-i) Specify who was blinded, and who wasn't

Specify who was blinded, and who wasn't. Usually, in web-based trials it is not possible to blind the participants [1, 3] (this should be clearly acknowledged), but it may be possible to blind outcome assessors, those doing data analysis or those administering co-interventions (if any).

	1	2	3	4	5	
subitem not at all important	○	○	○	○	○	essential

Does your paper address subitem 11a-i? *

Copy and paste relevant sections from the manuscript (include quotes in quotation marks "like this" to indicate direct quotes from your manuscript), or elaborate on this item by providing additional information not in the ms, or briefly explain why the item is not applicable/relevant for your study

Not applicable for the MRT because all participants received treatment at certain times, so it was not possible to blind them.

11a-ii) Discuss e.g., whether participants knew which intervention was the “intervention of interest” and which one was the “comparator”

Informed consent procedures (4a-ii) can create biases and certain expectations - discuss e.g., whether participants knew which intervention was the “intervention of interest” and which one was the “comparator”.

	1	2	3	4	5	
subitem not at all important	○	○	○	○	○	essential

Does your paper address subitem 11a-ii?

Copy and paste relevant sections from the manuscript (include quotes in quotation marks "like this" to indicate direct quotes from your manuscript), or elaborate on this item by providing additional information not in the ms, or briefly explain why the item is not applicable/relevant for your study

Participants did not know. All participants received the intervention of interest during some weeks of the study.

11b) If relevant, description of the similarity of interventions

(this item is usually not relevant for ehealth trials as it refers to similarity of a placebo or sham intervention to a active medication/intervention)

Does your paper address CONSORT subitem 11b? *

Copy and paste relevant sections from the manuscript (include quotes in quotation marks "like this" to indicate direct quotes from your manuscript), or elaborate on this item by providing additional information not in the ms, or briefly explain why the item is not applicable/relevant for your study

Not applicable.

12a) Statistical methods used to compare groups for primary and secondary outcomes

NPT: When applicable, details of whether and how the clustering by care providers or centers was addressed

Does your paper address CONSORT subitem 12a? *

Copy and paste relevant sections from the manuscript (include quotes in quotation marks "like this" to indicate direct quotes from your manuscript), or elaborate on this item by providing additional information not in the ms, or briefly explain why the item is not applicable/relevant for your study

Yes
"To estimate the coefficients, we use a multi-categorical extension of the weighted and centered least squares estimator described in [35]. The estimation method provides asymptotically unbiased estimates of the causal effect moderation of interest. The method also protects against potential misspecification of terms not interacted with treatment (\alpha_0X_t+\alpha_1M_t). The method assesses the uncertainty of the coefficient estimates using robust standard error estimation—the “sandwich” estimator [36]—to account for correlation between outcomes over time"

12a-i) Imputation techniques to deal with attrition / missing values

Imputation techniques to deal with attrition / missing values: Not all participants will use the intervention/comparator as intended and attrition is typically high in ehealth trials. Specify how participants who did not use the application or dropped out from the trial were treated in the statistical analysis (a complete case analysis is strongly discouraged, and simple imputation techniques such as LOCF may also be problematic [4]).

	1	2	3	4	5	
subitem not at all important	○	○	○	○	○	essential

Does your paper address subitem 12a-i? *

Copy and paste relevant sections from the manuscript (include quotes in quotation marks "like this" to indicate direct quotes from your manuscript), or elaborate on this item by providing additional information not in the ms, or briefly explain why the item is not applicable/relevant for your study

Yes
"Missingness occurred throughout the trial due to interns not completing self-reported mood survey or not wearing Fitbits. Multiple imputation [38], a robust method for dealing with missing data, was used to impute missing data at the daily level. Due to the complexity of the trial design and data structure, our imputation method combines imputation methods for longitudinal data [39] and sequentially randomized trials [40]. Results were aggregated across 20 imputed data sets using Rubin's rules [38], [41]. We also assessed the sensitivity of the conclusions to the imputation method. See Supplement Section 2 for further details on the missingness and sensitivity analysis results. "

12b) Methods for additional analyses, such as subgroup analyses and adjusted analyses

Does your paper address CONSORT subitem 12b? *

Copy and paste relevant sections from the manuscript (include quotes in quotation marks "like this" to indicate direct quotes from your manuscript), or elaborate on this item by providing additional information not in the ms, or briefly explain why the item is not applicable/relevant for your study

Yes, this is done in the supplement under "Additional Analyses". The techniques were similar to the main paper.

X26) REB/IRB Approval and Ethical Considerations [recommended as subheading under "Methods"] (not a CONSORT item)

X26-i) Comment on ethics committee approval

	1	2	3	4	5	
subitem not at all important	○	○	○	○	○	essential

Does your paper address subitem X26-i?

Copy and paste relevant sections from the manuscript (include quotes in quotation marks "like this" to indicate direct quotes from your manuscript), or elaborate on this item by providing additional information not in the ms, or briefly explain why the item is not applicable/relevant for your study

Yes, "UM IRB Protocol #HUM00033029"

x26-ii) Outline informed consent procedures

Outline informed consent procedures e.g., if consent was obtained offline or online (how? Checkbox, etc.?), and what information was provided (see 4a-ii). See [6] for some items to be included in informed consent documents.

	1	2	3	4	5	
subitem not at all important	○	○	○	○	○	essential

Does your paper address subitem X26-ii?

Copy and paste relevant sections from the manuscript (include quotes in quotation marks "like this" to indicate direct quotes from your manuscript), or elaborate on this item by providing additional information not in the ms, or briefly explain why the item is not applicable/relevant for your study

Yes "Participants were recruited through emails, which were sent to future interns from 47 different recruitment institutions between April 1, 2018 and June 25, 2018. The recruitment institutions comprised both medical schools, where emails were sent to all graduates, and residency locations, where emails were sent to all incoming interns. 5,233 future interns received the initial email inviting them to participate in the study. 2,134 (41%) interns downloaded the study app, completed the consent form, and filled out the baseline survey sometime before June 25, 2018. The study app and study participation were restricted to interns using an iPhone, the phone brand used by a majority of interns. The 2,134 interns received a Fitbit Charge 2. Of the 2,134, 1,565 interns (73%) were randomly selected to participate in the MRT (see Supplement Section 1 for an explanation of this initial randomization). These 1,565 interns were randomized according to Figure 2. Interns were incentivized to participate in the study by receiving the Fitbit wearable and up to $125, distributed five times throughout the year ($25 each time) based on continued participation."

X26-iii) Safety and security procedures

Safety and security procedures, incl. privacy considerations, and any steps taken to reduce the likelihood or detection of harm (e.g., education and training, availability of a hotline)

	1	2	3	4	5	
subitem not at all important	○	○	○	○	○	essential

Does your paper address subitem X26-iii?

Copy and paste relevant sections from the manuscript (include quotes in quotation marks "like this" to indicate direct quotes from your manuscript), or elaborate on this item by providing additional information not in the ms, or briefly explain why the item is not applicable/relevant for your study

This was not done.

RESULTS

13a) For each group, the numbers of participants who were randomly assigned, received intended treatment, and were analysed for the primary outcome

NPT: The number of care providers or centers performing the intervention in each group and the number of patients treated by each care provider in each center

Does your paper address CONSORT subitem 13a? *

Copy and paste relevant sections from the manuscript (include quotes in quotation marks "like this" to indicate direct quotes from your manuscript), or elaborate on this item by providing additional information not in the ms, or briefly explain why the item is not applicable/relevant for your study

Yes, "Participants were recruited through emails, which were sent to future interns from 47 different recruitment institutions between April 1, 2018 and June 25, 2018. The recruitment institutions comprised both medical schools, where emails were sent to all graduates, and residency locations, where emails were sent to all incoming interns. 5,233 future interns received the initial email inviting them to participate in the study. 2,134 (41%) interns downloaded the study app, completed the consent form, and filled out the baseline survey sometime before June 25, 2018. The study app and study participation were restricted to interns using an iPhone, the phone brand used by a majority of interns. The 2,134 interns received a Fitbit Charge 2. Of the 2,134, 1,565 interns (73%) were randomly selected to participate in the MRT (see Supplement Section 1 for an explanation of this initial randomization). These 1,565 interns were randomized according to Figure 2. Interns were incentivized to participate in the study by receiving the Fitbit wearable and up to $125, distributed five times throughout the year ($25 each time) based on continued participation."

13b) For each group, losses and exclusions after randomisation, together with reasons

Does your paper address CONSORT subitem 13b? (NOTE: Preferably, this is shown in a CONSORT flow diagram) *

Copy and paste relevant sections from the manuscript (include quotes in quotation marks "like this" to indicate direct quotes from your manuscript), or elaborate on this item by providing additional information not in the ms, or briefly explain why the item is not applicable/relevant for your study

Yes "Participants were recruited through emails, which were sent to future interns from 47 different recruitment institutions between April 1, 2018 and June 25, 2018. The recruitment institutions comprised both medical schools, where emails were sent to all graduates, and residency locations, where emails were sent to all incoming interns. 5,233 future interns received the initial email inviting them to participate in the study. 2,134 (41%) interns downloaded the study app, completed the consent form, and filled out the baseline survey sometime before June 25, 2018. The study app and study participation were restricted to interns using an iPhone, the phone brand used by a majority of interns. The 2,134 interns received a Fitbit Charge 2. Of the 2,134, 1,565 interns (73%) were randomly selected to participate in the MRT (see Supplement Section 1 for an explanation of this initial randomization). These 1,565 interns were randomized according to Figure 2. Interns were incentivized to participate in the study by receiving the Fitbit wearable and up to $125, distributed five times throughout the year ($25 each time) based on continued participation."

13b-i) Attrition diagram

Strongly recommended: An attrition diagram (e.g., proportion of participants still logging in or using the intervention/comparator in each group plotted over time, similar to a survival curve) or other figures or tables demonstrating usage/dose/engagement.

	1	2	3	4	5	
subitem not at all important	○	○	○	○	○	essential

Does your paper address subitem 13b-i?

Copy and paste relevant sections from the manuscript or cite the figure number if applicable (include quotes in quotation marks "like this" to indicate direct quotes from your manuscript), or elaborate on this item by providing additional information not in the ms, or briefly explain why the item is not applicable/relevant for your study

No, the attrition diagram was unnecessary and didn't provide anything necessary. Everything was described here:
"Participants were recruited through emails, which were sent to future interns from 47 different recruitment institutions between April 1, 2018 and June 25, 2018. The recruitment institutions comprised both medical schools, where emails were sent to all graduates, and residency locations, where emails were sent to all incoming interns. 5,233 future interns received the initial email inviting them to participate in the study. 2,134 (41%) interns downloaded the study app, completed the consent form, and filled out the baseline survey sometime before June 25, 2018. The study app and study participation were restricted to interns using an iPhone, the phone brand used by a majority of interns. The 2,134 interns received a Fitbit Charge 2. Of the 2,134, 1,565 interns (73%) were randomly selected to participate in the MRT (see Supplement Section 1 for an explanation of this initial randomization). These 1,565 interns were randomized according to Figure 2. Interns were incentivized to participate in the study by receiving the Fitbit wearable and up to $125, distributed five times throughout the year ($25 each time) based on continued participation."

14a) Dates defining the periods of recruitment and follow-up

Does your paper address CONSORT subitem 14a? *

Copy and paste relevant sections from the manuscript (include quotes in quotation marks "like this" to indicate direct quotes from your manuscript), or elaborate on this item by providing additional information not in the ms, or briefly explain why the item is not applicable/relevant for your study

Yes "Participants were recruited through emails, which were sent to future interns from 47 different recruitment institutions between April 1, 2018 and June 25, 2018. The recruitment institutions comprised both medical schools, where emails were sent to all graduates, and residency locations, where emails were sent to all incoming interns. 5,233 future interns received the initial email inviting them to participate in the study. 2,134 (41%) interns downloaded the study app, completed the consent form, and filled out the baseline survey sometime before June 25, 2018. The study app and study participation were restricted to interns using an iPhone, the phone brand used by a majority of interns. The 2,134 interns received a Fitbit Charge 2. Of the 2,134, 1,565 interns (73%) were randomly selected to participate in the MRT (see Supplement Section 1 for an explanation of this initial randomization). These 1,565 interns were randomized according to Figure 2. Interns were incentivized to participate in the study by receiving the Fitbit wearable and up to $125, distributed five times throughout the year ($25 each time) based on continued participation."

14a-i) Indicate if critical “secular events” fell into the study period

Indicate if critical “secular events” fell into the study period, e.g., significant changes in Internet resources available or “changes in computer hardware or Internet delivery resources”

	1	2	3	4	5	
subitem not at all important	○	○	○	○	○	essential

Does your paper address subitem 14a-i?

Copy and paste relevant sections from the manuscript (include quotes in quotation marks "like this" to indicate direct quotes from your manuscript), or elaborate on this item by providing additional information not in the ms, or briefly explain why the item is not applicable/relevant for your study

Not applicable.

14b) Why the trial ended or was stopped (early)

Does your paper address CONSORT subitem 14b? *

Copy and paste relevant sections from the manuscript (include quotes in quotation marks "like this" to indicate direct quotes from your manuscript), or elaborate on this item by providing additional information not in the ms, or briefly explain why the item is not applicable/relevant for your study

Not applicable. It ended at the intended time.

15) A table showing baseline demographic and clinical characteristics for each group

NPT: When applicable, a description of care providers (case volume, qualification, expertise, etc.) and centers (volume) in each group

Does your paper address CONSORT subitem 15? *

Copy and paste relevant sections from the manuscript (include quotes in quotation marks "like this" to indicate direct quotes from your manuscript), or elaborate on this item by providing additional information not in the ms, or briefly explain why the item is not applicable/relevant for your study

Yes, but not in a table as it was quite brief:
"Of the 1,565 interns in the MRT, 56% were female, 49% had previously experienced an episode of depression. The interns represented 321 different residency locations and 42 specialties. The study interns' baseline information closely resembled the known characteristics of the general medical intern population [19]. Throughout the trial, we measured intern mood valence, steps, and nightly sleep. Summaries of the weekly-level averages of those data can be found in Table 2."

15-i) Report demographics associated with digital divide issues

In ehealth trials it is particularly important to report demographics associated with digital divide issues, such as age, education, gender, social-economic status, computer/Internet/ehealth literacy of the participants, if known.

	1	2	3	4	5	
subitem not at all important	○	○	○	○	○	essential

Does your paper address subitem 15-i? *

Copy and paste relevant sections from the manuscript (include quotes in quotation marks "like this" to indicate direct quotes from your manuscript), or elaborate on this item by providing additional information not in the ms, or briefly explain why the item is not applicable/relevant for your study

Yes, but not in a table as it was quite brief:
"Of the 1,565 interns in the MRT, 56% were female, 49% had previously experienced an episode of depression. The interns represented 321 different residency locations and 42 specialties. The study interns' baseline information closely resembled the known characteristics of the general medical intern population [19]. Throughout the trial, we measured intern mood valence, steps, and nightly sleep. Summaries of the weekly-level averages of those data can be found in Table 2."

16) For each group, number of participants (denominator) included in each analysis and whether the analysis was by original assigned groups

16-i) Report multiple "denominators" and provide definitions

Report multiple "denominators" and provide definitions: Report N's (and effect sizes) "across a range of study participation [and use] thresholds" [1], e.g., N exposed, N consented, N used more than x times, N used more than y weeks, N participants "used" the intervention/comparator at specific pre-defined time points of interest (in absolute and relative numbers per group). Always clearly define "use" of the intervention.

	1	2	3	4	5	
subitem not at all important	○	○	○	○	○	essential

Does your paper address subitem 16-i? *

Copy and paste relevant sections from the manuscript (include quotes in quotation marks "like this" to indicate direct quotes from your manuscript), or elaborate on this item by providing additional information not in the ms, or briefly explain why the item is not applicable/relevant for your study

Yes, "Participants were recruited through emails, which were sent to future interns from 47 different recruitment institutions between April 1, 2018 and June 25, 2018. The recruitment institutions comprised both medical schools, where emails were sent to all graduates, and residency locations, where emails were sent to all incoming interns. 5,233 future interns received the initial email inviting them to participate in the study. 2,134 (41%) interns downloaded the study app, completed the consent form, and filled out the baseline survey sometime before June 25, 2018. The study app and study participation were restricted to interns using an iPhone, the phone brand used by a majority of interns. The 2,134 interns received a Fitbit Charge 2. Of the 2,134, 1,565 interns (73%) were randomly selected to participate in the MRT (see Supplement Section 1 for an explanation of this initial randomization). These 1,565 interns were randomized according to Figure 2. Interns were incentivized to participate in the study by receiving the Fitbit wearable and up to $125, distributed five times throughout the year ($25 each time) based on continued participation"

16-ii) Primary analysis should be intent-to-treat

Primary analysis should be intent-to-treat, secondary analyses could include comparing only "users", with the appropriate caveats that this is no longer a randomized sample (see 18-i).

	1	2	3	4	5	
subitem not at all important	○	○	○	○	○	essential

Does your paper address subitem 16-ii?

Copy and paste relevant sections from the manuscript (include quotes in quotation marks "like this" to indicate direct quotes from your manuscript), or elaborate on this item by providing additional information not in the ms, or briefly explain why the item is not applicable/relevant for your study

Yes, no users were dropped from the study. Intend to treat was used. "Participants were recruited through emails, which were sent to future interns from 47 different recruitment institutions between April 1, 2018 and June 25, 2018. The recruitment institutions comprised both medical schools, where emails were sent to all graduates, and residency locations, where emails were sent to all incoming interns. 5,233 future interns received the initial email inviting them to participate in the study. 2,134 (41%) interns downloaded the study app, completed the consent form, and filled out the baseline survey sometime before June 25, 2018. The study app and study participation were restricted to interns using an iPhone, the phone brand used by a majority of interns. The 2,134 interns received a Fitbit Charge 2. Of the 2,134, 1,565 interns (73%) were randomly selected to participate in the MRT (see Supplement Section 1 for an explanation of this initial randomization). These 1,565 interns were randomized according to Figure 2. Interns were incentivized to participate in the study by receiving the Fitbit wearable and up to $125, distributed five times throughout the year ($25 each time) based on continued participation"

17a) For each primary and secondary outcome, results for each group, and the estimated effect size and its precision (such as 95% confidence interval)

Does your paper address CONSORT subitem 17a? *

Copy and paste relevant sections from the manuscript (include quotes in quotation marks "like this" to indicate direct quotes from your manuscript), or elaborate on this item by providing additional information not in the ms, or briefly explain why the item is not applicable/relevant for your study

Yes

"Primary Aim

We conclude that previous week's average daily self-reported mood valence is a statistically significant negative moderator of the effect of notifications on

average daily self-reported mood valence. The estimate for the moderation is -0.052 (SE = 0.014, P = .001).

Figure 3 plots the estimated treatment effect at various values of the moderator. Figure 3 shows that the effect of notifications (compared to no notifications) was positive for weeks when previous mood was low, but negative for weeks when previous mood was high. For example, when previous week's average daily mood was 3, we estimate that a week of notifications increased an intern's average daily mood by 0.19 (effect size = 0.14). However, when previous week's average daily mood was 9, we estimate that a week of notifications decreased an intern's average daily mood by 0.12 (effect size = -0.08).

Exploratory Sub-Aim

For each notification category, we plotted the estimated treatment effect at various values of the moderator. Essentially, we broke apart the moderation effect in Figure 3 into the 3 categories of notifications. The result is shown in Figure 4. We included the line for general notifications from Figure 3 for reference. Figure 4 demonstrates that the moderation by previous week's average daily mood was similar for all 3 notification categories.

When previous week's average daily mood was 3, we estimated that a week of mood, activity, and sleep notifications increased an intern's average daily mood by 0.19, 0.16, 0.23 (effect sizes = 0.13, 0.11, 0.16), respectively. When previous week's average daily mood was 9, we estimated that a week of mood, activity, and sleep notifications decreased an intern's average daily mood by 0.12, 0.14, 0.09 (effect sizes = -0.08, -0.10, -0.06), respectively.

Secondary Aim 1

We conclude that previous week's average daily step count is a statistically significant negative moderator of the effect of activity notifications on average daily steps. The estimate for the moderation is -0.039 (SE = 0.015, P = .01).

Figure 5 plots the estimated treatment effect at various values of the moderator. In Figure 5, for interpretability, we re-transformed the moderation effect back

In Figure 5, for interpretability, we re-transformed the moderation effect back from the analysis scale (square root) to the original scale. We see from Figure 5 that the effect of activity notifications (compared to no notifications) was positive for weeks when previous steps were low, but negative for weeks when previous steps were high. For example, when previous week's average daily step count was 5,625, we estimate that a week of activity notifications increased an intern's average daily step count by 165 steps (effect size = 0.05). However, when previous week's average daily step count was 12,100, we estimate that a week of activity notifications decreased an intern's average daily step count by 60 steps (effect size = -0.02).

Secondary Aim 2

We conclude that previous week's average daily sleep is a statistically significant negative moderator of the effect of sleep notifications on average daily sleep. The estimate for the moderation is -0.075 (SE = 0.018, $P < .001$).

Figure 6 plots the estimated treatment effect at various values of the moderator. Again, we re-transformed the moderation effect back from analysis scale (square root) to the original scale. Also, for interpretability, the x-axis is on the hourly scale, while the y-axis is on the minute scale. We see from Figure 6 that the effect of sleep notifications (compared to no notifications) was positive for weeks when previous sleep was low, but negative for weeks when previous sleep was high. For example, when previous week's average daily sleep was 5 hours, we estimate that a week of sleep notifications increased an intern's average daily sleep by 8 minutes (effect size = 0.11). However, when previous week's average daily sleep was 8 hours, we estimate that a week of sleep notifications decreased an intern's average daily sleep by 5 minutes (effect size = -0.07).

Additional Analyses

Section 1 of the Supplement contains detailed results on additional analyses, including analysis of non-moderated main effects, changes in effects over time, effects of life insights and tips, effects on long-term PHQ-9 scores, and analysis of baseline moderators. There is evidence of a negative effect of (general)

notifications on mood. There is also evidence of a positive effect of activity notifications on step counts and sleep notifications on sleep duration. All of these effect sizes, however, are small. There is not strong evidence that these effects change over time. There is minor evidence that tips perform better than life insights on improving step count and sleep duration. We did not see any effects on long-term mental health outcomes. We saw some evidence of non-linear moderation for the primary and secondary aims. The non-linear moderator analysis suggested that, when the moderators are high, the treatment effect on sleep hours and step count is close to 0 (as opposed to negative). Finally, we found that baseline variables, such as gender and depression history, were weak moderators of notification effects, demonstrating the value of personalizing intervention delivery on real-time data.
"

17a-i) Presentation of process outcomes such as metrics of use and intensity of use

In addition to primary/secondary (clinical) outcomes, the presentation of process outcomes such as metrics of use and intensity of use (dose, exposure) and their operational definitions is critical. This does not only refer to metrics of attrition (13-b) (often a binary variable), but also to more continuous exposure metrics such as “average session length”. These must be accompanied by a technical description how a metric like a “session” is defined (e.g., timeout after idle time) [1] (report under item 6a).

	1	2	3	4	5	
subitem not at all important	○	○	○	○	○	essential

Does your paper address subitem 17a-i?

Copy and paste relevant sections from the manuscript (include quotes in quotation marks "like this" to indicate direct quotes from your manuscript), or elaborate on this item by providing additional information not in the ms, or briefly explain why the item is not applicable/relevant for your study

Not applicable. Use was not a concern. The supplement contains information on missing data.

17b) For binary outcomes, presentation of both absolute and relative effect sizes is recommended

Does your paper address CONSORT subitem 17b? *

Copy and paste relevant sections from the manuscript (include quotes in quotation marks "like this" to indicate direct quotes from your manuscript), or elaborate on this item by providing additional information not in the ms, or briefly explain why the item is not applicable/relevant for your study

Not applicable.

18) Results of any other analyses performed, including subgroup analyses and adjusted analyses, distinguishing pre-specified from exploratory

Does your paper address CONSORT subitem 18? *

Copy and paste relevant sections from the manuscript (include quotes in quotation marks "like this" to indicate direct quotes from your manuscript), or elaborate on this item by providing additional information not in the ms, or briefly explain why the item is not applicable/relevant for your study

Found in supplement under additional analyses.

18-i) Subgroup analysis of comparing only users

A subgroup analysis of comparing only users is not uncommon in ehealth trials, but if done, it must be stressed that this is a self-selected sample and no longer an unbiased sample from a randomized trial (see 16-iii).

	1	2	3	4	5	
subitem not at all important	○	○	○	○	○	essential

Does your paper address subitem 18-i?

Copy and paste relevant sections from the manuscript (include quotes in quotation marks "like this" to indicate direct quotes from your manuscript), or elaborate on this item by providing additional information not in the ms, or briefly explain why the item is not applicable/relevant for your study

Not applicable.

19) All important harms or unintended effects in each group

(for specific guidance see CONSORT for harms)

Does your paper address CONSORT subitem 19? *

Copy and paste relevant sections from the manuscript (include quotes in quotation marks "like this" to indicate direct quotes from your manuscript), or elaborate on this item by providing additional information not in the ms, or briefly explain why the item is not applicable/relevant for your study

No applicable

19-i) Include privacy breaches, technical problems

Include privacy breaches, technical problems. This does not only include physical “harm” to participants, but also incidents such as perceived or real privacy breaches [1], technical problems, and other unexpected/unintended incidents. “Unintended effects” also includes unintended positive effects [2].

	1	2	3	4	5	
subitem not at all important	○	○	○	○	○	essential

Does your paper address subitem 19-i?

Copy and paste relevant sections from the manuscript (include quotes in quotation marks "like this" to indicate direct quotes from your manuscript), or elaborate on this item by providing additional information not in the ms, or briefly explain why the item is not applicable/relevant for your study

Yes
"There were also a couple technological errors that occurred throughout the trial. There were 8 days (out of the 182 total days) where, due to server issues, no notifications were sent to any subject. Also, the weekly randomization to a notification category occurred without replacement, as opposed to with replacement as originally intended."

19-ii) Include qualitative feedback from participants or observations from staff/researchers

Include qualitative feedback from participants or observations from staff/researchers, if available, on strengths and shortcomings of the application, especially if they point to unintended/unexpected effects or uses. This includes (if available) reasons for why people did or did not use the application as intended by the developers.

	1	2	3	4	5	
subitem not at all important	○	○	○	○	○	essential

Does your paper address subitem 19-ii?

Copy and paste relevant sections from the manuscript (include quotes in quotation marks "like this" to indicate direct quotes from your manuscript), or elaborate on this item by providing additional information not in the ms, or briefly explain why the item is not applicable/relevant for your study

Not applicable.

DISCUSSION

22) Interpretation consistent with results, balancing benefits and harms, and considering other relevant evidence

NPT: In addition, take into account the choice of the comparator, lack of or partial blinding, and unequal expertise of care providers or centers in each group

22-i) Restate study questions and summarize the answers suggested by the data, starting with primary outcomes and process outcomes (use)

Restate study questions and summarize the answers suggested by the data, starting with primary outcomes and process outcomes (use).

	1	2	3	4	5	
subitem not at all important	○	○	○	○	○	essential

Does your paper address subitem 22-i? *

Copy and paste relevant sections from the manuscript (include quotes in quotation marks "like this" to indicate direct quotes from your manuscript), or elaborate on this item by providing additional information not in the ms, or briefly explain why the item is not applicable/relevant for your study

Yes
"Through this MRT of an mHealth push notification intervention, we found that the effects of notifications were negatively moderated by the subject's previous measurement of the outcome of interest. Specifically, we found that previous mood negatively moderated the effect of notifications on mood, previous step count negatively moderated the effect of activity notifications on step count, and previous sleep duration negatively moderated the effect of sleep notifications on sleep duration."

22-ii) Highlight unanswered new questions, suggest future research

Highlight unanswered new questions, suggest future research.

	1	2	3	4	5	
subitem not at all important	○	○	○	○	○	essential

Does your paper address subitem 22-ii?

Copy and paste relevant sections from the manuscript (include quotes in quotation marks "like this" to indicate direct quotes from your manuscript), or elaborate on this item by providing additional information not in the ms, or briefly explain why the item is not applicable/relevant for your study

Yes
"In addition to the research aims for future iterations of the IHS, assessing the value of mHealth interventions and delivery timing in other highly stressed populations is beneficial for understanding the generality of these results. Also, developing additional mHealth interventions beyond push notifications (e.g., combining with mindfulness apps) could provide a greater overall benefit."

"The IHS is an annual study which continues each year with a new cohort of interns [20]. Consistent with the MOST framework [49], [50], this provides multiple trial phases to continually update, optimize, and test interventions, and confirm findings from previous cohorts. Starting in the fall of 2019, we will run another study to test new hypotheses with improved interventions. Using the results and conclusions drawn from this study, in 2019 we plan to do the following:

1. Introduce tailored messages which are tailored based on an intern's previous mood, activity, and sleep [47], [48]. For people with high previous measurements, the messages will be framed towards maintenance of healthy behavior, not improvement. The cutoffs that define 'high' and 'low' scores will be based on data collected from the 2018 study.
2. Improved missing data protocol and incentive structure to reduce frequency of missingness.
3. Collect work schedule information. This information will be used to compare message efficacy between work days and days off.
4. Provide notification text within the app, as opposed to only on the phone lock screen, in order to give interns more opportunities to read the notifications.

"

20) Trial limitations, addressing sources of potential bias, imprecision, and, if relevant, multiplicity of analyses

20-i) Typical limitations in ehealth trials

Typical limitations in ehealth trials: Participants in ehealth trials are rarely blinded. Ehealth trials often look at a multiplicity of outcomes, increasing risk for a Type I error. Discuss biases due to non-use of the intervention/usability issues, biases through informed consent procedures, unexpected events.

	1	2	3	4	5	
subitem not at all important	○	○	○	○	○	essential

Does your paper address subitem 20-i? *

Copy and paste relevant sections from the manuscript (include quotes in quotation marks "like this" to indicate direct quotes from your manuscript), or elaborate on this item by providing additional information not in the ms, or briefly explain why the item is not applicable/relevant for your study

Yes
"
The main findings of the IHS MRT are partially sensitive to the imputation method used for dealing overcoming missing data (see Supplement Section 2). The conclusions of the primary aim and secondary aim 2 are not sensitive. The conclusion of secondary aim 1 (the negative moderation of the activity notification effect by previous step count), however, is sensitive to the imputation method.

The results of the IHS MRT may not extrapolate to other populations because medical interns are different from the general population in average education level and socioeconomic status. Within the population of medical interns, sampling bias may still exist as the study's interns self-selected into the study.

Daily work schedules were not reliably measured in this study. Prior studies [45], [46] have found that mHealth message effectiveness varies between weekdays and weekends, suggesting that future studies should assess work schedule as a potential moderator.

The primary outcome for the study, mood valence, was self-reported. In future studies, developing and using a passively-collected objective measurement of depression could be beneficial for improving validity and reducing missing data.

We did not have message tailoring in this study. Currently, the message framing and wording was the same, no matter the intern's current behavior. The messages (see Table 1) are framed towards improving mood, sleep, and activity. This framing may be frustrating to an intern who already has high mood or sufficient sleep/activity. Tailoring the wording of the messages [47], [48] could potentially eliminate the negative effect of messages when previous mood, sleep, or activity is high.

There were also a couple technological errors that occurred throughout the trial. There were 8 days (out of the 182 total days) where, due to server issues, no notifications were sent to any subject. Also, the weekly randomization to a notification category occurred without replacement, as opposed to with replacement as originally intended.
"

21) Generalisability (external validity, applicability) of the trial findings

NPT: External validity of the trial findings according to the intervention, comparators, patients, and care providers or centers involved in the trial

21-i) Generalizability to other populations

Generalizability to other populations: In particular, discuss generalizability to a general Internet population, outside of a RCT setting, and general patient population, including applicability of the study results for other organizations

	1	2	3	4	5	
subitem not at all important	○	○	○	○	○	essential

Does your paper address subitem 21-i?

Copy and paste relevant sections from the manuscript (include quotes in quotation marks "like this" to indicate direct quotes from your manuscript), or elaborate on this item by providing additional information not in the ms, or briefly explain why the item is not applicable/relevant for your study

Yes
"

The results of the IHS MRT may not extrapolate to other populations because medical interns are different from the general population in average education level and socioeconomic status. Within the population of medical interns, sampling bias may still exist as the study's interns self-selected into the study.
"

21-ii) Discuss if there were elements in the RCT that would be different in a routine application setting

Discuss if there were elements in the RCT that would be different in a routine application setting (e.g., prompts/reminders, more human involvement, training sessions or other co-interventions) and what impact the omission of these elements could have on use, adoption, or outcomes if the intervention is applied outside of a RCT setting.

	1	2	3	4	5	
subitem not at all important	○	○	○	○	○	essential

Does your paper address subitem 21-ii?

Copy and paste relevant sections from the manuscript (include quotes in quotation marks "like this" to indicate direct quotes from your manuscript), or elaborate on this item by providing additional information not in the ms, or briefly explain why the item is not applicable/relevant for your study

Not applicable

OTHER INFORMATION

23) Registration number and name of trial registry

Does your paper address CONSORT subitem 23? *

Copy and paste relevant sections from the manuscript (include quotes in quotation marks "like this" to indicate direct quotes from your manuscript), or elaborate on this item by providing additional information not in the ms, or briefly explain why the item is not applicable/relevant for your study

Yes "NCT03972293"

24) Where the full trial protocol can be accessed, if available

Does your paper address CONSORT subitem 24? *

Cite a Multimedia Appendix, other reference, or copy and paste relevant sections from the manuscript (include quotes in quotation marks "like this" to indicate direct quotes from your manuscript), or elaborate on this item by providing additional information not in the ms, or briefly explain why the item is not applicable/relevant for your study

Yes, the supplement contains further details on the analysis. The registration has more details on the protocol

25) Sources of funding and other support (such as supply of drugs), role of funders

Does your paper address CONSORT subitem 25? *

Copy and paste relevant sections from the manuscript (include quotes in quotation marks "like this" to indicate direct quotes from your manuscript), or elaborate on this item by providing additional information not in the ms, or briefly explain why the item is not applicable/relevant for your study

Yes
"NIH grants R01MH101459, P30CA046592 (National Cancer Institute, Cancer Center Support Grant (CCSG) Development Funds from Rogel Cancer Center), a grant from Michigan Institute for Data Science, an investigator award from Michigan Precision Health Initiative."

X27) Conflicts of Interest (not a CONSORT item)

X27-i) State the relation of the study team towards the system being evaluated

In addition to the usual declaration of interests (financial or otherwise), also state the relation of the study team towards the system being evaluated, i.e., state if the authors/evaluators are distinct from or identical with the developers/sponsors of the intervention.

	1	2	3	4	5	
subitem not at all important	○	○	○	○	○	essential

Does your paper address subitem X27-i?

Copy and paste relevant sections from the manuscript (include quotes in quotation marks "like this" to indicate direct quotes from your manuscript), or elaborate on this item by providing additional information not in the ms, or briefly explain why the item is not applicable/relevant for your study

Yes, this is present under the intern health study website: "https://www.srijan-sen-lab.com/intern-health-study"

About the CONSORT EHEALTH checklist

As a result of using this checklist, did you make changes in your manuscript? *

- ○ yes, major changes
- ◉ yes, minor changes
- ○ no

What were the most important changes you made as a result of using this checklist?

Added more details on a few items

How much time did you spend on going through the checklist INCLUDING making changes in your manuscript *

5 hours

As a result of using this checklist, do you think your manuscript has improved? *

- ○ yes
- ◉ no
- ○ Other:

Would you like to become involved in the CONSORT EHEALTH group?

This would involve for example becoming involved in participating in a workshop and writing an "Explanation and Elaboration" document

- ○ yes
- ◉ no
- ○ Other:

Any other comments or questions on CONSORT EHEALTH

Your answer

STOP - Save this form as PDF before you click submit

To generate a record that you filled in this form, we recommend to generate a PDF of this page (on a Mac, simply select "print" and then select "print as PDF") before you submit it.

When you submit your (revised) paper to JMIR, please upload the PDF as supplementary file.

Don't worry if some text in the textboxes is cut off, as we still have the complete information in our database. Thank you!

Final step: Click submit !

Click submit so we have your answers in our database!

SUBMIT

Never submit passwords through Google Forms.

This content is neither created nor endorsed by Google. Report Abuse - Terms of Service

Google Forms
